# New Paradigm for Nano–Bio Interactions: Multimolecular Assembly of a Prototypical Disordered Protein with Ultrasmall Nanoparticles

**DOI:** 10.1021/acs.nanolett.2c02902

**Published:** 2022-11-08

**Authors:** Giovanna Viola, Carlo Giorgio Barracchia, Roberto Tira, Francesca Parolini, Giulia Leo, Massimo Bellanda, Francesca Munari, Stefano Capaldi, Mariapina D’Onofrio, Michael Assfalg

**Affiliations:** † Department of Biotechnology, University of Verona, 37134 Verona, Italy; ‡ Department of Chemistry, University of Padova, 35131 Padova, Italy

**Keywords:** intrinsically disordered proteins, NMR spectroscopy, protein aggregation, protein−nanoparticle interaction, ultrasmall nanoparticles

## Abstract

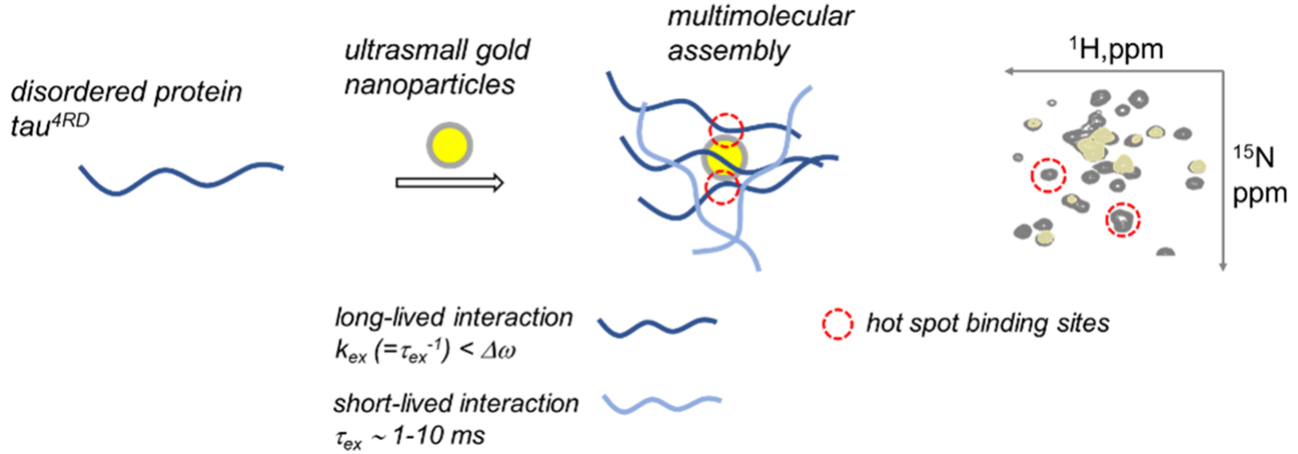

Understanding the interactions between nanoparticles (NPs) and proteins is crucial for the successful application of NPs in biological contexts. Protein adsorption is dependent on particle size, and protein binding to ultrasmall (1–3 nm) NPs is considered to be generally weak. However, most studies have involved structured biomacromolecules, while the interactions of ultrasmall NPs with intrinsically disordered proteins (IDPs) have remained elusive. IDPs are abundant in eukaryotes and found to associate with NPs intracellularly. As a model system, we focused on ultrasmall gold nanoparticles (usGNPs) and tau, a cytosolic IDP associated with Alzheimer’s disease. Using site-resolved NMR, steady-state fluorescence, calorimetry, and circular dichroism, we reveal that tau and usGNPs form stable multimolecular assemblies, representing a new type of nano–bio interaction. Specifically, the observed interaction hot spots explain the influence of usGNPs on tau conformational transitions, with implications for the intracellular targeting of aberrant IDP aggregation.

The successful development of nanoparticle (NP)-based tools for use in biological contexts requires a thorough understanding of the nano–bio interactions.^1^ Despite the inherent complexity of interfaces, significant progress has been made in the description of the physicochemical interactions between nanomaterials and biological components.^2^ A major result is the observation that most NPs readily interact with biomolecules that adsorb on their surface, forming a biomolecular corona which influences their biodistribution and bio-activity.^3,4^

Among NPs, ultrasmall NPs (usNPs), usually defined as particles with core size in the range 1–3 nm, present attractive features for biomedical purposes, including efficient renal clearance, limited accumulation in the liver, in vivo tumor accumulation, enhanced cell and nuclear penetration, and capability to cross the blood–brain barrier.^5−8^ Ultrasmall gold NPs (usGNPs) have the advantage of controllable synthesis, ease of surface modification, emergent optical properties, and low toxicity.^9,10^ Therefore, usGNPs show great promise for applications in biosensing, cellular imaging, drug delivery, and disease therapy.^11,12^

A systematic investigation of protein adsorption to citrate-stabilized GNPs upon exposure to biological media provided clear evidence that the characteristics of the protein corona are particle-size-dependent.^13^ Thus, large NPs become coated by a thick, multilayered corona, while medium-sized NPs display a near-single dense protein corona layer, and small NPs exhibit an incomplete corona. In the ultrasmall scale, the nature of protein–NP interactions differs from the case of larger NPs, being characterized by transient particle–biomolecule associations and by the absence of hard coatings.^14,15^ Indeed, usNPs are of comparable size or smaller than common proteins and may display macromolecule-like or even drug-like features. The formed supramolecular species can be defined as usNP/protein complexes.^16^

A significant fraction of proteins interacting with NPs in a living system is constituted by macromolecules of defined tertiary structure, including albumin, immunoglobulins, and others that are abundant in peripheral biofluids.^17,18^ However, a recent study revealed that NP-associated intracellular proteomes are enriched in intrinsically disordered, RNA-processing proteins, suggesting that conformational disorder facilitates the binding of proteins to NPs.^19^ In addition to RNA-binding proteins, biologically active intrinsically disordered proteins (IDPs) and proteins containing disordered regions (IDRs) are abundant in eukaryotic proteomes.^20^ Besides being involved in key biological functions, an increasing number of IDPs are found to undergo aberrant aggregation, under defined conditions, and are associated with irreversible neurodegenerative diseases.^21^ In this context, the binding to NPs could provide the means to redirect aggregation pathways and mitigate the formation of neurotoxic assemblies, opening the way to new therapeutic strategies.^22,23^

Classical NPs offer large surface areas for accommodating disordered polypeptides, whose conformational adaptability allows establishing a large number of noncovalent interactions. The adsorption of amyloidogenic proteins and peptides onto NPs and the consequences on protein fibrillation have been extensively explored.^22,24^ At the macroscopic level, NPs were found to either accelerate or retard protein fibril formation, depending on surface properties.^23,25^ In the ultrasmall regime, the interactions of IDPs with NPs remain poorly characterized.^26−28^

Herein, we focus on the interaction of usGNPs with a prototypical IDP: the aggregation-prone, four-repeat domain of tau (tau^4RD^, Supporting Information Figure S1A). Tau is a cytosolic IDP that supports neuronal cell function and that, in pathological conditions, transitions from a soluble monomeric state to oligomers and fibrils (hallmark aggregates in Alzheimer’s disease, AD).^29,30^ A high number of basic amino acid residues mediate the interaction of tau^4RD^ with biological and exogeneous anionic surfaces (microtubules, lipid membranes, NPs). To characterize the mode of interaction between usGNPs and tau^4RD^, we applied gel electrophoresis, steady-state fluorescence spectroscopy, isothermal titration calorimetry, circular dichroism spectroscopy, and site-resolved solution NMR. We further explored the influence of usGNPs on disease-related protein conformational transitions.

We prepared dihydrolipoic acid (DHLA)-capped usGNPs (ligand structure is displayed in Figure S1B) with a previously described one-pot synthetic approach.^31^ DHLA-capped usGNPs (hereafter referred to as usGNPs for simplicity) exhibit limited ligand desorption due to the strong Au–S bonds and form stable colloids in aqueous solution due to electrostatic repulsion between carboxylates.^31,32^ The colloidal solution of usGNPs appeared pale brown and displayed bright red luminescence on exposure to UV light (Figure S2A). The particles were characterized by UV–visible absorption spectroscopy, transmission electron microscopy (TEM), dynamic light scattering (DLS), and ^1^H NMR spectroscopy (Figure S2B–H). The mean core size was ca. 1.9 nm, and the ζ-potential value in neutral solution was ζ = −36 ± 6 mV.

To investigate protein binding to usGNPs, we first applied agarose gel electrophoresis. UsGNPs are expected to migrate toward the positive electrode. Conversely, tau^4RD^ is a lysine-rich, highly basic polypeptide that displays positive ζ potential (ζ = 22 ± 1 mV, p*I* = 9.7) and is predicted to migrate toward the cathode. In this work, we used a cysteine-free protein variant (hereafter, tau^4RD^) to avoid uncontrolled formation of Au–S and S–S bonds. The observed progressive decrease of usGNP electrophoretic mobility toward the anode on increasing protein concentration (Figure 1A) likely originated from both an increased size and a surface charge variation determined by the association of usGNPs with tau^4RD^.

**Figure 1:**
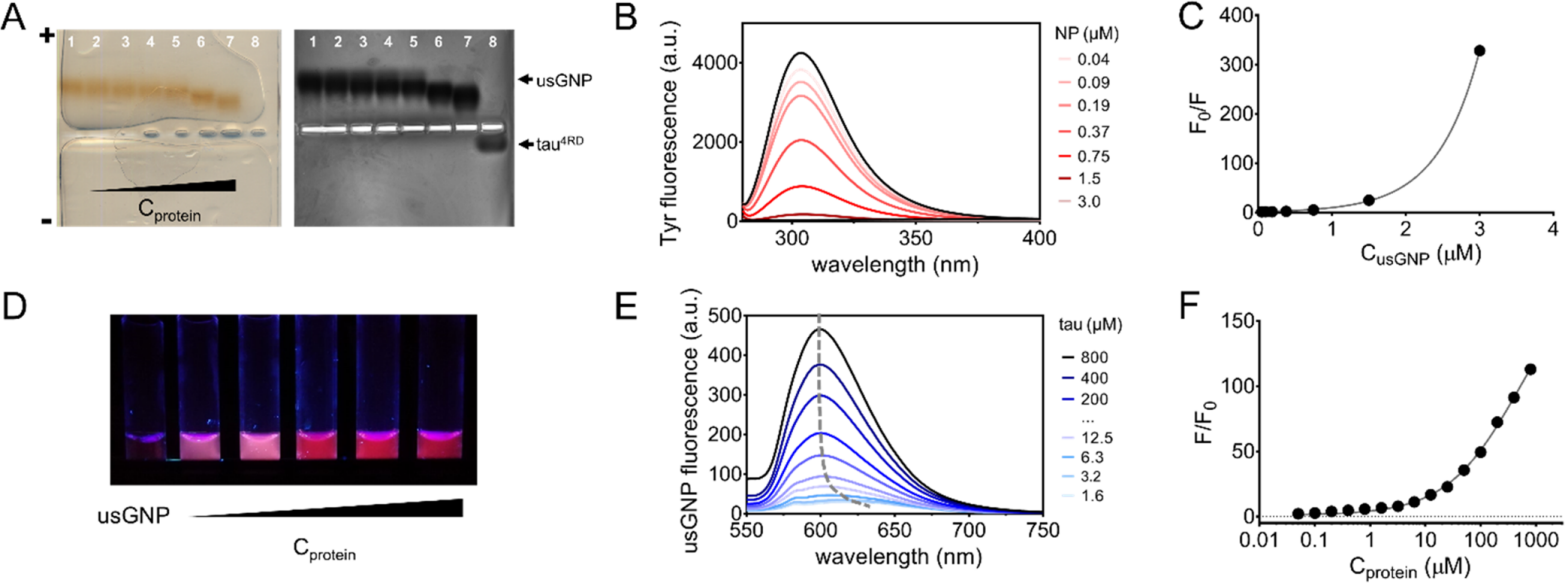
Gel electrophoresis and photophysical measurements. (A) Agarose native gel electrophoresis: left, unstained; right, Coomassie staining. Lanes 1–7 were loaded with 6.5 μM usGNPs; lanes 2–8 contained tau^4RD^ at concentrations 3.25, 6.5, 13, 19.5, 43.5, and 65 μM; all components were dissolved in 0.5% TBE, pH 9 (1% agarose, 50 V, 40 min). (B) Tyrosine fluorescence emission spectra (λ_ex_ = 270 nm) measured on 6 μM tau^4RD^ in the presence of usGNPs at varying concentration. The spectrum of the free protein is shown in black (λ_max_ = 304 nm). (C) Relative Tyr fluorescence intensity as a function of usGNP concentration (Stern–Volmer plot). The solid line corresponds to an exponential function fit. (D) Colloidal solutions of 0.5 μM usGNPs in the presence of varying concentration of protein, visualized under UV lamp. (E) Fluorescence emission spectra (λ_ex_ = 530 nm) of 0.25 μM usGNP in the presence of tau^4RD^ at varying concentration. The dashed line indicates the shift of the peak maximum. (F) Relative usGNP fluorescence intensity as a function of protein concentration (logarithmic scale). The solid line corresponds to the best-fit curve (Hill function).

We further examined the effect of the interaction on the photophysical properties of protein and usGNPs. The intrinsic fluorescence of the protein, due to the single fluorescent residue Tyr310, was effectively quenched on addition of usGNPs (Figure 1B), suggesting that the repeat region R3 closely approached the particle surface. The nonlinear dependence of the relative fluorescence intensity, *F*_0_/*F*, on usGNPs concentration (Figure 1C) indicates that the quenching mechanism was not of a single type (dynamic or static). Previous work established that fluorescence quenching of organic dyes by usGNPs lacking a surface plasmon band was dominated by nanometal surface energy transfer.^33^

As noted earlier, the photoluminescence of usGNPs is generally affected by protein binding.^34^ Here, we observed a progressive enhancement of intrinsic luminescence on increasing the concentration of tau^4RD^ (Figure 1D), with up to 20-fold higher intensity for the highest concentration of tau^4RD^ considered, and a blue shift of the emission band maximum (up to 40 nm) (Figure 1E). The latter observation indicates that adsorbed protein molecules reduced the polarity of the local dielectric environment at the particle surface and suggests that tau^4RD^ has a greater capability to shield the usGNP surface from the solvent, compared to compact globular proteins.^34^ The fluorescence intensity did not show saturation behavior in the investigated concentration range (Figure 1F); therefore, an accurate quantitative analysis of the experimental curve was not possible. Nonetheless, a tentative fit of the data with a Hill model yielded a Hill coefficient *n* = 0.66, suggestive of anti-cooperative binding.

To gain insight into the mechanism of association, we performed isothermal titration calorimetry (ITC) experiments. The calorimetric response obtained by titrating tau^4RD^ into a solution of usGNPs appeared nonmonotonic, with a net heat release in the first half and endothermic behavior in the second half of the titration (Figures 2A,S3). Interestingly, the titration of another IDP, α-synuclein, into usGNPs generated a different calorimetric profile, lacking net endothermic signals (Figure S4) but also reflecting the occurrence of multiple processes during titration. The calorimetric curves for both protein systems were ionic-strength-dependent, displaying reduced heat exchange at high salt concentration (Figures 2B,S3, and S4), thus pointing to a contribution of electrostatic interactions to the underlying phenomena. The effect was particularly evident in the second part of the process: at high salt concentration, the positive hump in the calorimetric profile of tau^4RD^ and the negative shoulder in that of α-synuclein were attenuated. In the case of tau^4RD^ in salt-containing solutions, the early titration heat signals departed from the general trend of the following titration points (note that solution buffers for injectant and analyte were identical) (Figures 2B, S3). This behavior was not observed for α-synuclein (Figure S4), indicating that the effect was protein-specific. Calorimetric data for tau^4RD^ were analyzed with a two-sets-of-sites binding model to estimate thermodynamic quantities (Tables 1, S1). In the absence of NaCl, binding was strong for both sets, driven by enthalpy in the first and by entropy in the second event. The number of involved binding sites was larger for the second set than for the first set. The energetic terms and affinity constants were reduced in the presence of 100 mM NaCl. The favorable enthalpic term likely resulted from strong electrostatic attraction between the negatively charged usGNPs and the polycationic protein, while the favorable entropic component of the second event may reflect, among various possible contributing factors, the conformational disorder of bound protein molecules. Thus, the proposed model identified two main modes of association of tau^4RD^ with usNPs: the one characterized by greater affinity may correspond to unhindered docking of protein molecules to free surface areas on usNPs, while the lower affinity mode may correspond to the binding to already coated usNPs. The occurrence of two distinct types of interaction recalls the concept of hard and soft corona described for larger NPs. However, differently from a typical hard corona where folded proteins form a densely packed and sterically defined adlayer, tau molecules bound to usGNPs are probably best described as a “fuzzy” assembly, where fuzziness refers to the conformational heterogeneity of bound-state ensembles of IDPs observed in many protein–protein complexes.^35^ On binding to usNPs, IDPs will engage small contact regions, by contrast larger NPs may accommodate long polypeptide stretches and multiple regions. Thus, IDPs interacting with larger NPs can form fuzzy complexes^36^ as well as more static and compact protein coronas.^37^

**Figure 2:**
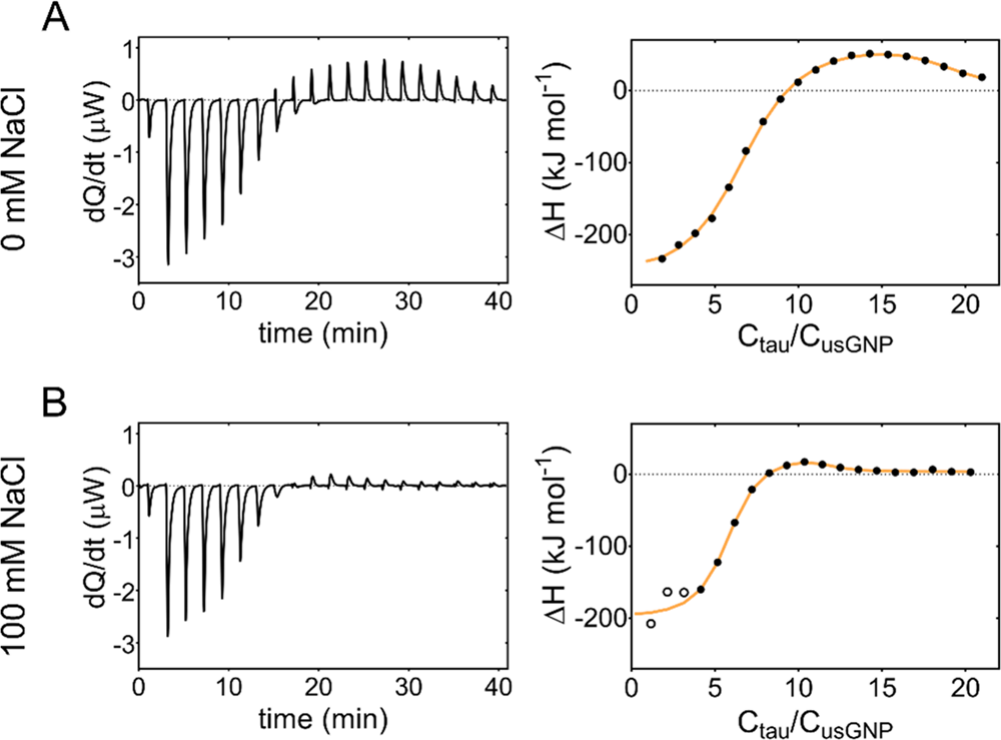
Energetics of the interaction between tau^4RD^ and usGNPs. Isothermal titration calorimetry data obtained on titrating tau^4RD^ into usGNPs, in the presence of 0 mM (A) and 100 mM (B) NaCl (see Figure S3 for additional NaCl concentration points). Left panels display corrected heat transfer rates; right panels display integrated heat plots. Orange lines are best-fit curves based on a two-sets-of-sites binding model; data displayed as empty circles were excluded from fitting.

**Table 1:** Thermodynamic Parameters for the Binding of tau^4RD^ to usGNPs^a^

NaCl	b	*K*_a_ (M^–1^)	Δ*G* (kJ mol^–1^)	Δ*H* (kJ mol^–1^)	–*T*Δ*S* (kJ mol^–1^)	*n*
0 mM	I	(6.1 ± 0.9) × 10^7^	–44.5 ± 0.4	–268 ± 5	224 ± 5	5.8 ± 0.1
	II	(2.7 ± 0.3) × 10^6^	–36.8 ± 0.3	74 ± 5	–110 ± 5	12.5 ± 0.1
100 mM	I	(8.1 ± 1.2) × 10^6^	–39.5 ± 0.4	–216 ± 20	177 ± 20	5.5 ± 0.8
	II	(3.1 ± 0.5) × 10^5^	–31.4 ± 0.4	65 ± 10	–96 ± 10	6 ± 3

a Given errors are from data fitting.

b I, first binding event; II, second binding event.

The disordered character of tau^4RD^ entails strong conformational plasticity and adaptability to binding surfaces.^23^ To obtain insight into protein structural changes resulting from adsorption to usGNPs, we performed circular dichroism (CD) measurements. The far-UV CD spectrum of tau^4RD^ free in solution featured a deep ellipticity minimum centered at 198 nm (Figure S5A), consistent with a prevalently unstructured state. The addition of usGNPs elicited the progressive reduction of signal ellipticity and a shift of the peak minimum toward longer wavelengths. We interpret these spectral changes as being due to a binding-induced redistribution of conformational states and formation of a heterogeneous ensemble of bound molecules with mixed-type local secondary structure elements.^38,39^ The peak wavelength change as a function of usGNP concentration was found to follow an apparent Langmuir-type dependence (Figure S5B), indicating saturable binding.

To identify the NP-binding site(s) on tau^4RD^, we performed site-resolved NMR spectroscopy experiments. Previous works demonstrated the power of NMR to elucidate the binding modes and dynamics of proteins, both folded and unstructured, on NP surfaces with single residue resolution.^36,40−47^ The ^1^H–^15^N HSQC spectrum of [^15^N]tau^4RD^ displays HN correlation signals for virtually all nonproline amino acid residues (Figures 3A,S6). After addition of small amounts of usGNPs, the position of individual peaks was minimally perturbed (for P:usGNP = 0.02, Δδ_HN_ were <0.015 ppm); by contrast signal intensities were significantly reduced (Figures 3A,B, S6). In the initial titration steps, no new signals appeared; however at higher usGNP concentration, a number of broad, low-intensity peaks became visible at different positions from those of the unbound protein (Figure 3A). This behavior, also in consideration of the binding strength determined by ITC, is consistent with the partitioning of protein molecules to a particle-bound state in a slow–intermediate exchange regime. The absence of sharp bound-state resonances can be attributed to multiple molecules interacting with each particle, forming assemblies with reduced rotational diffusion, and to structural disorder and dynamics of the bound-state conformational ensemble contributing to peak broadening. The residue-by-residue signal intensity attenuation was not uniform along the peptide sequence, with a similar profile at low (Figure 3B) and high ionic strength (Figure S7), indicating preferential involvement of discrete regions in binding to usGNPs. For example, the strong attenuations in segment 276–283, encompassing part of one hexapeptide motif (PHF6*, 275–280), suggest that this region represents a preferential anchoring site for usGNPs. Indeed, two adjacent lysine residues (Lys280, Lys281), a unique occurrence in the entire polypeptide, may promote the association via strong electrostatic attraction. Interestingly, intensity perturbations were also strong (>80%, Figure 3B) in the stretch 309–311, which belongs to the second hexapeptide motif (PHF6, 306-311) and terminates with Lys311, hinting at a central role of these motifs in the protein’s slow exchange dynamics.

**Figure 3:**
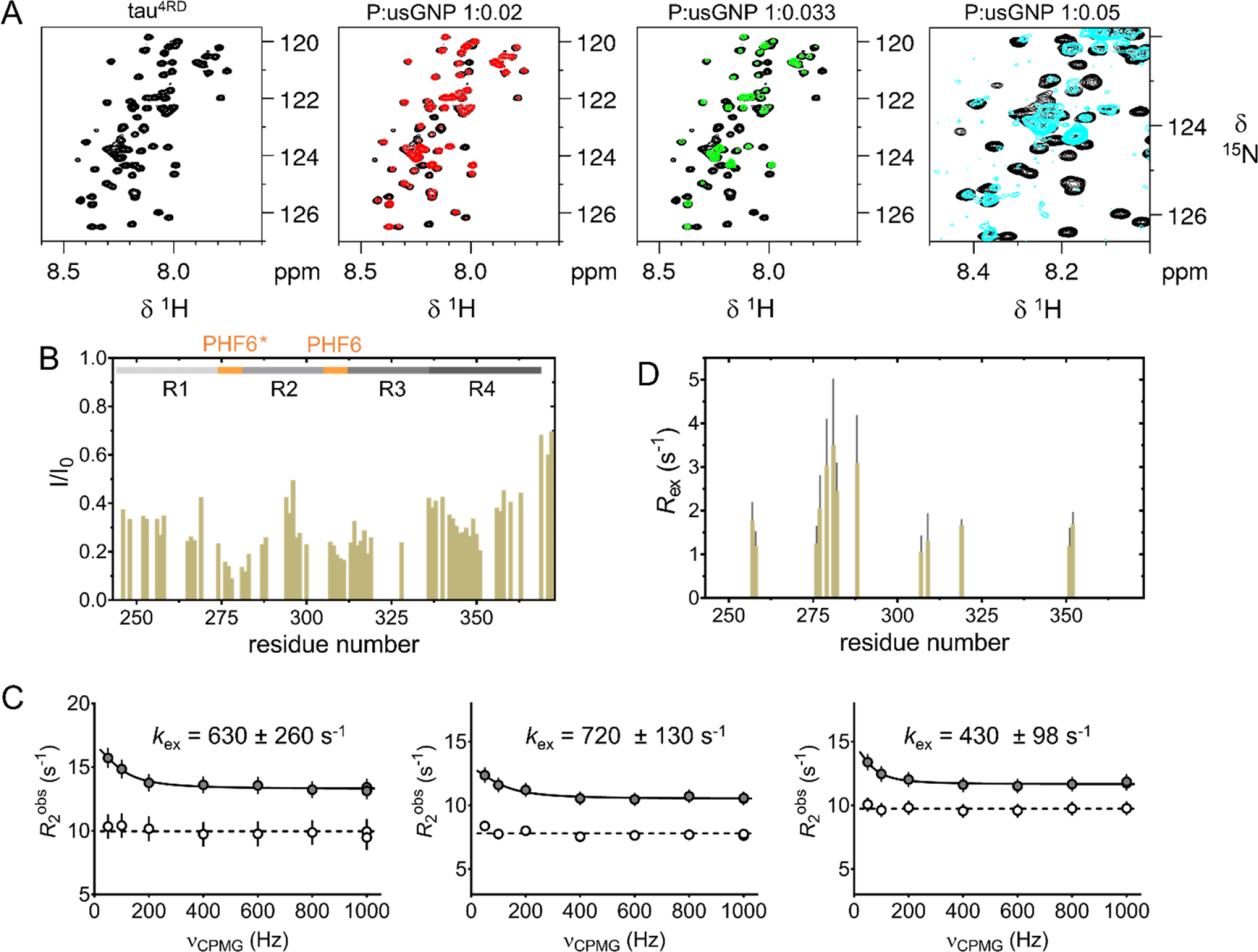
Mapping of contact sites and conformational dynamics. (A) Selected portions of HN-HSQC spectra (600 MHz) of 50 μM [^15^N]tau^4RD^ (black) in the absence (top left) or presence of usGNPs (colored maps overlaid on black) at the reported molar ratios. Red and green maps are shown in scale with black spectrum, the cyan map is displayed with increased intensity for better visualization of the peak position changes; the rightmost panel displays an enlarged view for better appreciation of peak details; and protein (P):usGNP molar ratios are indicated on top. (B) Residue-specific HSQC-peak intensity versus residue number. Peak intensities were measured on tau^4RD^ in the absence (*I*_0_) or presence (*I*) of usGNPs at a molar ratio P:usGNPs = 0.02; only isolated peaks were included in the analysis; the protein domain organization is schematized with bars of different gray shading for the four repeat motifs (R1–R4); and the hexapeptide motifs are indicated in orange. (C) Representative ^15^N-CPMG relaxation dispersion curves at 700 MHz spectrometer frequency observed for 200 μM [^15^N]tau^4RD^ in the absence (empty circles) or presence (filled circles) of 2 μM usGNPs (from left to right: Lys281, Leu282, Gln288) and 10 mM NaCl. Uncertainties were estimated from duplicate measurements; solid lines are the best-fit curves obtained by fitting the relaxation data to a two-state exchange model, the fitted exchange rate constants are reported; and dashed lines indicate the average *R*_2_^obs^ for no exchange. (D) Exchange contributions to relaxation rates obtained from relaxation dispersion experiments on samples containing tau^4RD^ and usGNPs. Gray bars are errors propagated from the uncertainties of fitted parameters.

To gain additional insight into the dynamics of tau^4RD^ in the presence of usGNPs, we carried out ^15^N-spin Carr–Purcell–Meiboom–Gill relaxation dispersion (CPMG-RD) experiments, which are sensitive to exchange processes in the 0.3–10 ms time window.^48^ Chemical exchange signal broadening, resulting from an enhanced relaxation rate (line width λ = 2*R*_2_^obs^, where *R*_2_^obs^ = *R*_2_^0^ + *R*_ex_), is modulated by the frequency of the CPMG pulse train (ν_CPMG_) applied during the *T*_CPMG_ relaxation delay of the NMR experiment. The analysis of *R*_2_^obs^ versus ν_CPMG_ dispersion curves provides information on the underlying dynamic process. The protein alone displayed virtually no relaxation dispersion, by contrast small but clear-cut RD effects for some residues were apparent in the presence of usGNPs (Figure 3C,D). Dispersion curves were analyzed with two-state exchange models, and all data were found to best fit to an exchange process in the fast-limit regime. This process likely corresponds to the weak binding of protein molecules to already coated NPs since the experiment was performed with protein in large excess. Observing the effects of the intermediate dynamics that characterizes the protein–NP interactions would require comparable concentrations of protein and NPs corresponding to conditions unsuitable for relaxation dispersion measurements. Relaxation dispersion originates from changes in the chemical environment at the observed site, and, interestingly, the largest chemical exchange contribution to transverse relaxation, *R*_ex_, was observed in the region 277–282 and for residue 288 (Figure 3D). We further note that the effective relaxation rate values at high CPMG field strength (ν_CPMG_ → ∞), *R*_2_^0^, corresponding to relaxation in the absence of exchange, were invariably larger for tau^4RD^ in the presence of usGNPs than for the protein alone. The residue-by-residue variation of Δ*R*_2_ (with/without usGNP, Figure S8), measured at a CPMG field of 595 Hz to suppress exchange-induced line broadening, reports on contributions to *R*_2_ from the NP-bound state.^49,50^ The patterned profile again indicates the prevalent involvement of the PHF6 regions in the assembly with usGNPs.

Following the observation that aggregation-prone hexapeptide motif regions were key mediators of binding to usGNPs in both slower and faster exchange regimes, we set to explore the influence of usGNPs on protein aggregation. The aggregation kinetics of tau^4RD^ was followed by monitoring the time-dependent fluorescence signal of thioflavin-T (ThT). The kinetic profile was sigmoidal-shaped and consistent with a macroscopic nucleation–growth mechanism (Figure 4A). The presence of low amounts of usGNPs (P:NP < 0.005) showed modest influence on the kinetics of the process, as evident from the little changes in transition midpoints compared to the particle-free sample. However, the addition of usGNPs at a P:NP ratio of 1:0.03 resulted in a significantly delayed aggregation (midpoint transition time constant, *t*_0.5_ = 30.1 ± 0.4 h versus 20.7 ± 0.1 h in the absence of NPs) and almost unchanged fibril elongation rate (elongation time constant, τ = 1.6 ± 0.3 h versus 1.4 ± 0.1 h). The fluorescence intensity at plateau decreased progressively on increasing the concentration of usGNPs until complete quenching in the case of a P:NP molar ratio of 1:0.1 (Figure 4A), possibly indicating the formation of smaller amounts of fibrils.

**Figure 4:**
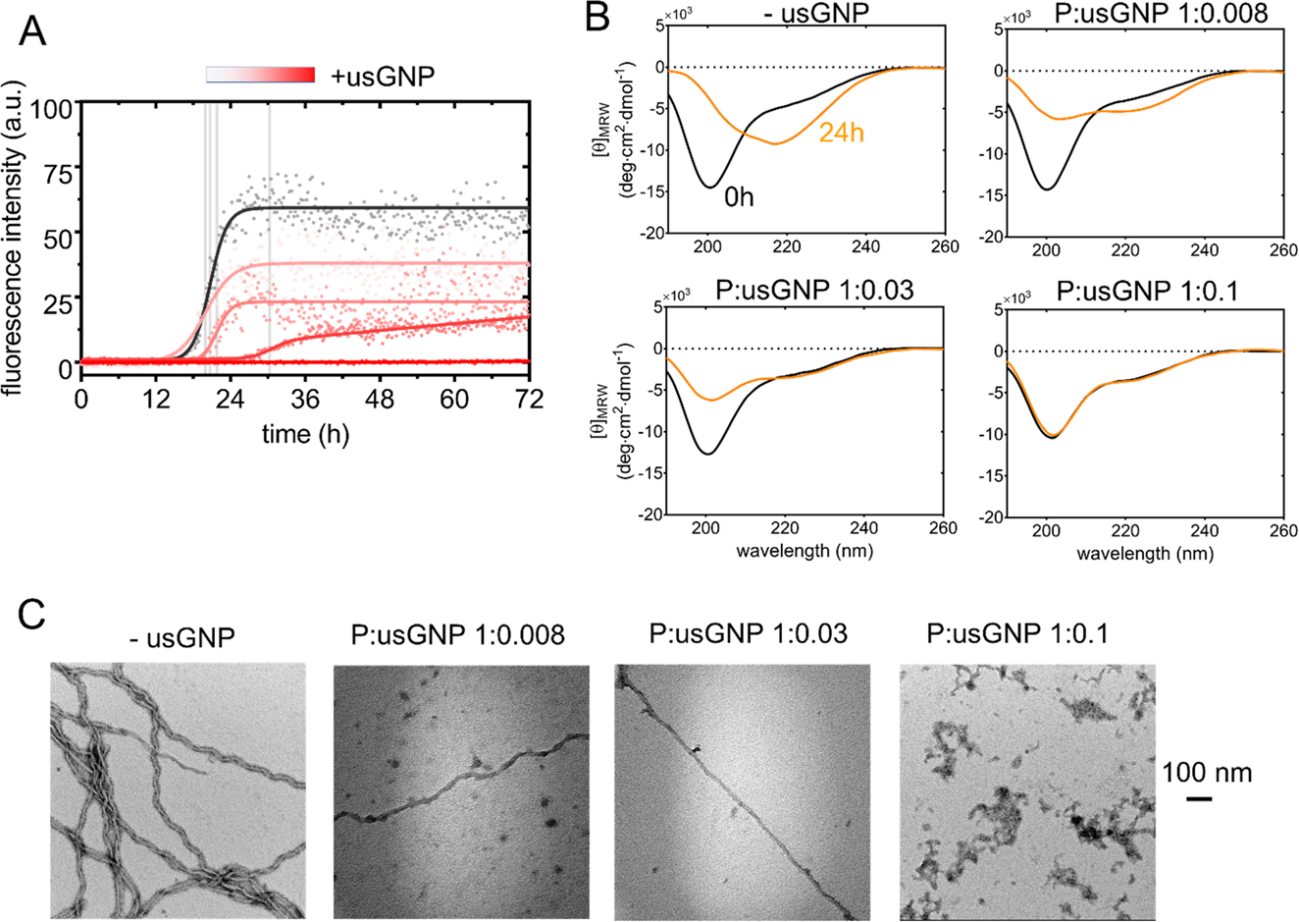
Protein conformational transitions and aggregation. (A) Aggregation kinetics monitored by ThT fluorescence. Measurements were performed on tau^4RD^ in the absence (gray dots) or presence (light-to-dark red dots) of usGNPs (P:usGNP molar ratios 1:0, 1:0.002, 1:0.004, 1:0.03, 1:0.1); solid lines correspond to the best-fit curves determined using an empirical sigmoid function; data represent the mean of five replicate measurements; and vertical gray lines indicate transition midpoints. (B) Far-UV CD spectra acquired on 6 μM tau^4RD^ after 0 h (black) and 24 h (brown) incubation, in the absence or presence of usGNPs at the indicated molar ratios. (C) Representative TEM images of tau^4RD^ samples after 48 h incubation in aggregating conditions, in the absence (left panel) or presence (remaining panels) of usGNPs (see Figure S9 for additional images). Scale bar is 100 nm.

The protein conformational transitions taking place during aggregation were further monitored by far-UV CD (Figure 4B). The spectrum of tau^4RD^ in the absence of usGNPs revealed a major change in secondary structure content after 24 h of incubation: the ellipticity minimum shifted to larger wavelengths (217 nm), consistent with the formation of β-structure, and a shoulder appeared around 207 nm, possibly corresponding to mixed-type secondary structures.^39^ In the presence of usGNPs at a P:NP ratio of 1:0.1, no conformational conversion was observed after the incubation period, while experiments conducted with lower amounts of particles revealed an intermediate situation with a smaller signal of the disordered protein and slightly increased ellipticity around ∼220 nm (Figure 4B). Thus, the CD data were consistent with the concentration-dependent trend observed in the ThT fluorescence assay. TEM analysis of samples incubated for 48 h showed that filamentous aggregates could form in the presence of low–intermediate concentrations of usGNPs but not when particles were added at a P:NP ratio of 1:0.1 (Figures 4C, S9). By coarse visual inspection of the micrographs, we found that the amount of fibrillar aggregates decreased with increasing usGNP concentration, confirming the inhibitory effect of usGNPs. The inhibitory action of usGNPs was retained at higher (near-physiological) ionic strength (Figure S7).

The possibility to use usNPs for modulating the conformational states of IDPs intracellularly stimulated us to verify two fundamental requirements for usGNPs: (i) their biocompatibility and (ii) their ability to get internalized into live neuronal cell models. To assess the biocompatibility, we evaluated the viability of two different human cell lines, the neuroglioma-derived H4swe and the nontumoral human embryonic kidney (HEK-293), after 72 h treatment with various concentrations of usGNPs. We observed no significant decrease in cell viability for both lines (Figure S10A), demonstrating that usGNP does not display acute cytotoxicity. Next, we monitored the cellular uptake and the internalization efficiency of usGNPs in H4swe cells by confocal microscopy. Since autofluorescence of usGNPs was insufficient to provide a clear signal at the used concentrations, we resorted to labeling the particles with cysteamine-FITC. Small granular aggregates were clearly visible in the cytoplasm of cells treated for 48 h with labeled nanoparticles (Figure S10B). Overall, these results indicate that usGNPs are internalized by the cells with little or no toxicity in the short term, making them suitable for in-cell applications. The observation that usGNPs concentrated intracellularly in discrete sites stimulated us to verify whether they localized in stress granules (SGs). These cytoplasmic membraneless organelles contain several RNA-binding proteins which are enriched for intrinsically disordered regions (IDRs) and thus could represent targets for usGNPs. Colocalization experiments showed that upon simple incubation, usGNPs did not apparently partition into SGs induced by arsenite treatment nor did they trigger SG formation (Figure S11). However, if cell membrane permeabilization was applied to facilitate particle uptake after SG induction, usGNPs distributed throughout the cell interior and partitioned into SGs (Figure S12), suggesting that usGNPs may interact with IDRs/IDPs in these organelles.

In conclusion, usNPs are attracting considerable interest for potential applications in the life sciences. The possibility to design usNPs that target biologically active macromolecules and modulate their function requires in-depth characterization of their interactions with biomolecules. It has been observed that particle size strongly influences NP–protein interactions and that protein binding to usNPs is generally weak, at least in the case of globular folded proteins. However, the nature of the interactions of usNPs with disordered proteins (IDPs) has been poorly investigated. In our study, we aimed at a detailed, submolecular level characterization of a prototypical system consisting of usGNPs and the disordered tau protein. We found that usGNPs engage the protein tau^4RD^ in the formation of stable (*K*_a_ = ∼ 10^7^ M^–1^) multimolecular assemblies (Figure 5), in contrast to the notion of weak protein association to usNPs. Additional short-lived (τ_ex_ = 1–10 ms) interactions were also detected, likely corresponding to binding events on the exterior of usGNP/tau complexes. The formed assemblies are best described as “fuzzy” complexes, with reference to bound-state conformational heterogeneity. Different from assemblies with larger NPs, the interaction of IDPs with usNPs cannot involve long polypeptide stretches and may not result in the formation of a sterically defined and compact protein corona. This type of assembly can affect biological behavior: specifically, by targeting disease-related protein aggregation sites of tau, usGNPs were found to act as aggregation inhibitors. Given the observation that usGNPs are not cytotoxic and are taken up by neuronal cells, they could find application for aggregation studies in both in vitro and cellular models of neurodegeneration.

**Figure 5:**
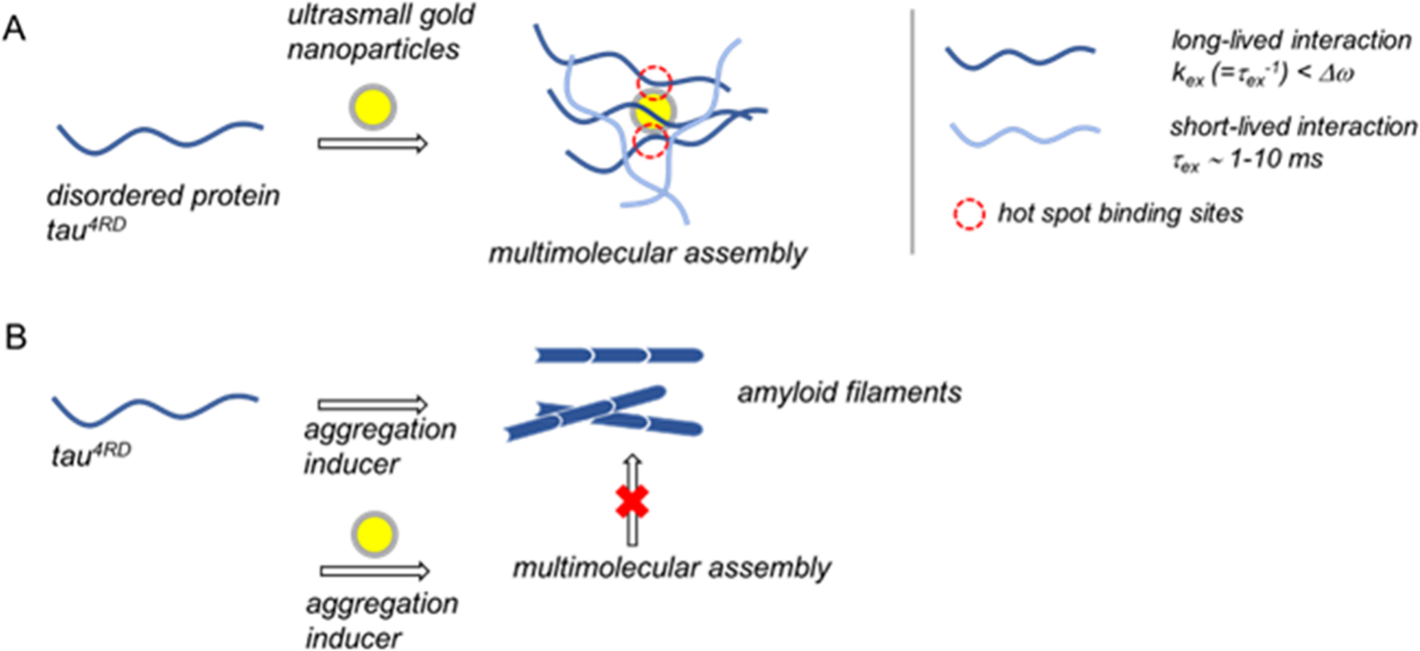
(A) Schematic representation of the multimolecular assembly formed by ultrasmall gold NPs and a prototypical intrinsically disordered protein, characterized by two distinct exchange regimes. (B) Schematic representation of the influence of usGNPs on disease-related amyloid deposition of tau^4RD^.

## Supporting Information Available

Experimental methods; Tables S1 and S2, binding parameters; Figure S1, schematic of molecules; Figure S2, characterization synthesized ultrasmall gold nanoparticles; Figure S3, ionic-strength thermal response; Figure S4, isothermal titration calorimetry; Figure S5, secondary structure perturbations; Figure S6, protein-observed NMR spectra; Figure S7, binding and aggregation; Figure S8, ^15^N-ΔR_2_ data; Figure S9, TEM images; Figure S10, biocompatibility and internalization; Figures S11 and S12, colocalization experiments (PDF)
