# Supplementary material for: New Paradigm for Nano–Bio Interactions: Multimolecular Assembly of a Prototypical Disordered Protein with Ultrasmall Nanoparticles
Source: Nano Lett. 2022 Nov 8;22(22):8875–82. doi: 10.1021/acs.nanolett.2c02902 (PMC9706667; doi:10.1021/acs.nanolett.2c02902)
Supplement: Supplementary file 1 — nl2c02902_si_001.pdf [file nl2c02902_si_001.pdf]

SUPPORTING INFORMATION

New Paradigm for Nano−Bio Interactions: Multimolecular Assembly of a Prototypical Disordered Protein with Ultrasmall Nanoparticles

Giovanna Viola,[a] Carlo Giorgio Barracchia,[a] Roberto Tira,[a] Francesca Parolini,[a] Giulia Leo,[a] Massimo Bellanda,[b] Francesca Munari,[a] Stefano Capaldi,[a] Mariapina D'Onofrio[a] and Michael Assfalg[a]

[a] Department of Biotechnology, University of Verona, 37134 Verona, Italy

[b] Department of Chemistry, University of Padova, 35131 Padova, Italy

CONTENT:

- Experimental methods

- Tables S1, S2

- Figures S1-S12

METHODS

Reagents

Tetrachloroauric acid trihydrate ($HAuCl_4$), lipoic acid (LA), and sodium borohydride ($NaBH_4$) were purchased from Alfa Aesar (MA, USA). Heparin and all chemical reagents were obtained from Sigma-Aldrich (St Louis, MO, USA). In all preparations, high-purity deionized water from a Millipore system was used.

Recombinant protein expression and purification

Recombinant tau^{4RD} (region Q244-E372 of human full-length tau, plus initial Met) was expressed and purified as described previously,[1] with the mutations C291A and C322A to avoid unintended disulfide bond formation (hereafter referred to as tau^{4RD} for simplicity). Briefly, the protein was expressed in BL21 (DE3) cells grown in LB medium at 37 °C for 5 h, with 0.5 mM IPTG. Protein purification was achieved by thermal treatment of the soluble bacterial extract (80–100 °C) and SP-ion exchange chromatography. To label tau^{4RD} with ^{15}N, the *E. coli* culture was grown in M9 minimal medium supplemented with $^{15}NH_4Cl$ (1 g/L). Purified tau^{4RD} products were dialyzed in the final buffer (10 mM phosphate buffer pH 7.4, hereafter called working buffer). Recombinant human α-synuclein was produced in *E. coli* BL21 (DE3) cells and purified as reported previously.[2]

Synthesis of lipoic acid-stabilized ultrasmall gold nanoparticles

For synthetic procedures, all glassware was cleaned with 70% HNO_3 and rinsed thoroughly in Millipore water prior to use. Dihydrolipoic acid(DHLA)-capped ultrasmall gold nanoparticles (usGNPs) were synthesized following a previously reported protocol.[3,4]. Briefly, 70 mg of lipoic acid were dissolved in 188.6 ml aqueous solution containing 484 µL NaOH (2 M), followed by addition of $HAuCl_4$ solution (1.96 mL, 2% by mass). After stirring for 5 min, an aqueous solution of $NaBH_4$ (4.06 mL, 50 mM) was slowly added drop by drop, under rapid stirring. The reaction was stopped after stirring overnight. The brownish solution was dialysed against ultrapure water, and then overnight against working buffer, using a 3.5 kDa molecular weight cut-off membrane. The solution was purified by centrifugal filtration, using Amicon Ultra (Merck, Millipore) centrifugal filters with a 10 kDa cut-off. The pale brown solution containing the usGNPs was stored at 4 °C for later use.

The nanoparticle concentration was estimated from the absorbance at 450 nm using the extinction coefficient (4.25×10^5) M^{-1} cm^{-1}, as reported by Haiss.[5] UV-vis absorption spectra were recorded with a NanoDrop™ 2000 Spectrophotometer (Thermofisher), using 1 cm path-length quartz cuvettes.

Cysteamine-FITC/DHLA-capped usGNPs were synthesized following the same protocol described above. [3,4] Cysteamine (0.05 M) was mixed with FITC 8 mg/ml in sodium carbonate buffer, pH 9.0, for 24 h at room temperature in the dark. The product was then mixed with lipoic acid in a molar ratio of 16:1.

Dynamic Light Scattering (DLS)

Hydrodynamic diameter and ζ-potential measurements were performed using a Zetasizer Nano ZS (Malvern Instruments, Malvern, UK) at room temperature, operating at λ = 633 nm and equipped with a back scattering detector of 173°. For determining size distribution by DLS, a solution of 13 µM usGNPs in working buffer was recorded in triplicate and the average of three measurements was plotted. For ζ-potential measurements, samples were loaded into a folded capillary cell and the voltage was automatically set to 148 V. The ζ-potential values were derived from the electrophoretic mobility by means of the Henry equation over five replicates.

Fluorescence Spectroscopy

Fluorescence measurements were performed on a Jasco FP-8500 spectrofluorometer (Jasco, Easton, MD, USA) using 1 cm path-length quartz cuvettes. The used concentrations of usGNPs and tau^{4RD} were such that the absorbance at the excitation wavelength was less than 0.05, to avoid self-absorption and inner filter effects.

Fluorescence emission spectra of usGNPs were recorded in the range of 550-750 nm using an excitation wavelength of 530 nm (slit width 5 nm). A fixed concentration of usGNPs (0.25 µM) was titrated with tau^{4RD} (800-0.05 µM). Samples were prepared in working buffer.

Tyrosine fluorescence measurements were performed on a Jasco FP-8500 spectrophotometer (Jasco, Easton, MD, USA). The excitation wavelength was 270 nm (slid width 10 nm) and emission spectra were collected in the range of 275-400 nm. A fixed concentration of tau^{4RD} (60 µM) was titrated with usGNPs (3-0.1 µM).

All experiments were performed at room temperature. Three spectra accumulations were averaged for each sample and the spectrum of the buffer was considered as a blank and subtracted.

Isothermal Titration Calorimetry (ITC)

ITC measurements were performed with a MicroCal PEAQ-ITC instrument (Malvern Panalytical, UK) at 25 °C. 1.5 µM of usGNPs were titrated with tau^{4RD} or α-synuclein (150 µM and 250 µM, respectively). Nineteen injections of 2 µL were performed, setting the stirring speed to 500 rpm, with 150 s interval between subsequent injections. For all titrations, samples were dialysed against the same working buffer. Number of sites (N), dissociation constant (K_D) and enthalpy change (ΔH) were the experimental parameters obtained by the best performing fitting model (two sets of sites). Data were analyzed by using the MicroCal PEAQ-ITC Analysis Software (Malvern). The titrations were repeated in the presence of different concentrations of NaCl (10, 50, 100 and 200 mM NaCl for tau^{4RD}, 10 and 200 mM NaCl for α-synuclein).

Nuclear Magnetic Resonance (NMR) spectroscopy

NMR spectra were recorded on a Bruker Avance IV 700 MHz spectrometer, equipped with a TCI cryoprobe, or on a Bruker Avance NEO 600 MHz spectrometer equipped with a Prodigy TCI cryoprobe. All the spectra were recorded at 10 °C.

One-dimensional ^{1}H spectra were acquired on DHLA-stabilized usGNPs (5 µM) and DHLA solutions, in working buffer and 10% D_2O, for general characterization. Experiments were performed using a standard pulse sequence incorporating the excitation sculpting water suppression scheme. A total of 8 transients were acquired over a spectral width of 9615 Hz and 32768 complex points with recycle delay of 1.7 s.

NMR titration experiments were run on samples with a protein concentration of 50 µM and usGNPs concentration in the range 0-5 µM, corresponding to protein/usGNPs molar ratios of: 10:1, 20:1, 25:1, 30:1, 40:1, 50:1, 60:1, and 80:1. The solutions were prepared in working buffer and 10% D_2O. One-dimensional ^{1}H-NMR experiments were acquired with 16 transients over a spectral width of 11363 Hz and 32768 complex points, with a recycle delay of 1.2 s. Typical two-dimensional ^{1}H-^{15}N HSQC spectra were acquired with a data matrix consisting of 2048 (F2, ^{1}H) x 256 (F1, ^{15}N) complex points, 12 scans, 1.2 s recycle delay, and spectral widths of 9615 (F2) x 1776 (F1) Hz.

Nuclear spin relaxation rate measurements were performed at 16.4 T magnetic field strength. Protein backbone ^{15}N-spin T_2 (=R_2^{-1}) spectra were recorded on 200 µM [^{15}N]tau^{4RD} in the absence or presence of usGNPs. Experiments were performed in gradient-selected sensitivity-enhanced mode and in interleaved fashion; matrix: 2048(^{1}H) × 128(^{15}N) complex data points for each relaxation delay; spectral widths: 13 (^{1}H) and 33 ppm (^{15}N). Water signal suppression was obtained with a flip-back pulse. The recycle delay was set to 3 s. T_2 relaxation delays were 0.016 (duplicate), 0.032, 0.064, 0.128, 0.192, 0.224, 0.272 (duplicate) s. The echo delay was 0.42 ms. Relaxation times T_2 were determined by fitting peak intensities to a single exponential decay.

Carr–Purcell Meiboom–Gill relaxation dispersion (CPMG-RD) experiments were performed using a constant-time relaxation-compensated pulse program (constant-time period, T_{CPMG}= 100 ms; CPMG frequencies, (ν_{CPMG} = 50, 100, 200, 400, 600, 800, 1000(duplicate) Hz). Two-dimensional data sets were acquired in an interleaved manner (inter-scan delay = 3 s; 256 increments in the nitrogen dimension). The transverse relaxation rate, R_2^{obs}, for each frequency point was obtained from the signal intensity measured at the end of the T_{CPMG} period according to:

$$R_2^{obs} = -\frac{\ln\left(I_{\nu\mathrm{CPMG}}/\mathrm{I}_0\right)}{T_{\mathrm{CPMG}}} \quad \text{Eq. 1}$$

where I_0 is the signal intensity measured in a reference spectrum lacking the CPMG period, and $I_{\nu CPMG}$ is the residual intensity at the end of the CPMG pulse sequence for a particular spin-lock frequency. R_2^{obs} uncertainties were estimated from duplicate measurements of one data point and were in most cases below 5%. The minimum error was set to 5% for all points.

Relaxation dispersion curves were modelled with a simple two-state exchange process in RING-NMR.[6] Fast-limit ($k_{ex} \gg \delta\omega$) and slow-limit ($k_{ex} \ll \delta\omega$) exchange models between the sites were considered, model selection was based on the Akaike information criterion. Dispersion curves were then fitted individually with the resulting fast-exchange model using GraphPad Prism 9 (GraphPad Software Inc., La Jolla, CA, USA). The fitting parameters were k_{ex}, R_2^0, and δ_{ppm}^{min}; the latter is a field-independent variable representing the chemical shift change expected for equal populations of the exchanging states. The exchange contributions to relaxation rates were calculated from the expression:

$$R_{ex} = \frac{\left(2\pi B_0 \delta_{ppm}^{min}\right)^2}{4k_{ex}} \qquad \text{Eq. 2}$$

R_{ex} uncertainties were obtained by error propagation from the fitted parameters.

NMR spectra were processed using TOPSPIN 4.1.1 (Bruker, Karlsruhe, Germany) and analyzed with NMRFAM SPARKY (T. D. Goddard and D. G. Kneller, University of California, San Francisco).

Far-UV Circular Dichroism (CD) spectroscopy

CD measurements were carried out on a Jasco J-1500 spectropolarimeter equipped with a Peltier type temperature-controlled cell holder (Jasco, Easton, MD, USA). Far-UV spectra (190–260 nm) were recorded in 0.1 cm cuvettes, at 25 °C, with a scan rate of 50 nm min^{-1}, a bandwidth of 1 nm, and an integration time of 2 s. Spectra were recorded on samples of tau^{4RD} in the absence or presence of usGNPs at various molar ratios. Five spectra accumulations were averaged for each sample and the spectrum of the buffer was considered as a blank and subtracted. The protein concentration was 6 µM.

Prior to running aggregation assays, solutions of tau^{4RD} were filtered through a 100 kDa cut-off filter (Sartorius Stedim Biotech GmbH, Göttingen, Germany) to remove pre-existing large oligomers and fibrils. To monitor time-dependent spectral changes, samples were incubated for 48 h at 30 °C. Aggregation reactions were prepared in 300 µL aqueous buffer (10 mM KPi, pH 7.4, protease inhibitors, in the absence or presence of 100 mM NaF) by mixing 50 µM tau^{4RD}, (0.4, 1.5, 5) µM usGNPs, and 50 µM heparin.

Data plots were generated with GraphPad Prism 9 (GraphPad Software Inc., La Jolla, CA, USA).

Thioflavin-T (ThT) aggregation assay

Solutions of tau^{4RD} were preliminarily filtered through a 100 kDa cut-off filter. The aggregation was induced by incubating the soluble protein in the presence of heparin at various concentrations of usGNPs; protein:NP was (10:0, 10:0.02, 10:0.04, 10:0.3, 10:1) µM in working buffer (in the absence or presence of 100 mM NaCl). Control reactions were carried out in the absence of usGNPs (protein:HEP was 10:10 µM).

The kinetics of aggregation was monitored by measuring the fluorescence of thioflavin-T (10 µM) added to each sample in a 96-well dark plate (100 µL final volume for each well). Fluorescence measurements were performed using a TECAN Infinite M200 Pro microplate reader (Tecan Group AG, Männedorf, Switzerland) at

30 °C for ca.72 h with cycles of 30 s of shaking (250 rpm, orbital) and 10 min of rest throughout the incubation. The fluorescence intensity was measured every 11 min (excitation, 450 nm; emission, 480 nm; bottom read). Error bars of fluorescence data correspond to standard deviations of at least four independent experiments.

Transmission Electron Microscopy (TEM)

For TEM measurements, samples were prepared as described for the ThT assay at the final volume of 100 µL and incubated at 30 °C for 48 h in static condition. Subsequently, 30 µL of aggregates samples (5 µM) in mQ H_2O were adsorbed onto 400 mesh holey film grids; after staining with 2% uranyl acetate (for 2 min), the sample was observed with a Tecnai G^2 (FEI) transmission electron microscope operating at 100 kV. Images were captured with a Veleta (Olympus Soft Imaging System, Münster, Germany) digital camera using FEI TIA acquisition software (Version 4.0).

Cellular viability and nanoparticle uptake assays

APP-swe expressing cells (H4-swe cells) and Hek-293 cells were cultured in a humidified atmosphere of 5% CO_2, and passaged in complete growth medium: Dulbecco's modified Eagle's medium (DMEM) High Glucose (Aurogene) containing 10% fetal bovine serum (FBS) (Aurogene) supplemented with 2 mM glutamine (Aurogene), 100 U/ml penicillin and 100 U/ml streptomycin (Aurogene). Once 70–80% confluence was reached, cells were collected using trypsin, washed, and counted.

usGNPs were dialyzed against phosphate-buffered saline (PBS) prior to administration to cells. For the treatment, FBS was replaced with 2% B27 Supplement. Cell viability after usGNPs treatment was evaluated by the reduction of the tetrazolium salt MTT 1-(4,5-Dimethylthiazol-2-yl)-3,5-diphenylformazan, Thiazolyl blue formazan Sigma Aldrich), following the manufacturer's protocol. Briefly, 10000 H4-swe cells/well were seeded in their exponential growth phase in a flat-bottomed 96-well plate and were incubated at 37 °C in a 5% CO_2 incubator. After 24 h, cells were treated with different concentrations of usGNPs (0.5, 0.1, 0.05, and 0.01 µM). After 72 h of treatment, cells were incubated with 0.5 mg/ml MTT for 3 h at 37 °C and insoluble formazan crystals were dissolved in 200 µl DMSO. Reduced MTT was evaluated by measuring the absorbance at 560 nm. Experiments were performed in triplicate on an Infinite M200 PRO fluorescence microplate reader (Tecan).

For usGNPs uptake experiments, human H4-swe cells were seeded on coverslip glasses in a 24-multiwell microplate. Cells were treated for 48 h with 0.5 µM FITC-usGNPs in complete medium (replacing 10% FBS with 2% B27 Supplement). After treatment, cells were washed with PBS and fixed with ice-cold 4% PFA in PBS for 15 min at room temperature. After blocking with 2% bovine serum albumin and 2% Fetal Goat Serum in PBS for 1 h at room temperature, cells were incubated with 2 µg/ml of anti β-tubulin III primary antibody (Merck) diluted in the blocking buffer for 2 h at room temperature. After 3 PBS washes, cells were incubated with 0.5 µg/ml anti-rabbit-Alexa Fluor 594 secondary antibody (Thermo Scientific) diluted in the blocking

buffer for 1 h at room temperature. After PBS washes, nuclei were stained with 1 μg/ml Hoechst diluted in PBS.

For stress granules (SG) colocalization experiments, H4-swe cells (104 cells/well) were seeded on coverslip glasses in a 24-multiwell microplate. Cells were treated overnight with 0.5 μM FITC-usGNP as described above. The next day, coverslips were washed twice with complete DMEM medium and incubated 1 h at 37 °C in presence or absence of 0.5 mM sodium arsenite (AS). Cells were washed three times with PBS, fixed with ice-cold 4% PFA for 20 min and permeabilized with 0.2% Triton X-100 in PBS. After blocking with 1% bovine serum albumin in PBS, cells were incubated for 2 h with CoraLite®594-conjugated G3BP1 monoclonal antibody (Proteintech) diluted in blocking solution. After 3× PBS washes, nuclei were stained with 1 μg/ml Hoechst diluted in PBS and coverslips were sealed with nail polish. Alternatively, live cells were first treated with 0.5 mM AS (1 h) and subsequently permeabilized with 0.01% Triton X-100 in HHBS [Hanks' Buffer with 20 mM Hepes] for 5 min. After 2 washes with HHBS, coverslips were incubated with 0.5 μM FITC-usGNP diluted in the same buffer at 37 °C for 30 min. After 3 washes with HHBS, cells were fixed and immunostained as described above.

Microscopy images were acquired using a Leica DM2500 fluorescence microscope or a confocal laser-scanning fluorescence microscope Leica TCS SP5 with a 63x HCX PL APO objective, and analyzed with LAS AF (Leica) and ImageJ software.

Table S1. Thermodynamic parameters for the binding of tau^{4RD} to usGNPs. Given uncertainties are from data fitting.

NaCl	*	K_a (M^{-1})	ΔG ($kJmol^{-1}$)	ΔH ($kJmol^{-1}$)	-TΔS ($kJmol^{-1}$)	n
0 mM	I)	$(6.1 \pm 0.9) \times 10^7$	-44.5 ± 0.4	-268 ± 5	224 ± 5	5.8 ± 0.1
	II)	$(2.7 \pm 0.3) \times 10^6$	-36.8 ± 0.3	74 ± 5	-110 ± 5	12.5 ± 0.1
10 mM	I)	$(1.3 \pm 0.1) \times 10^8$	-46.4 ± 0.2	-290 ± 6	244 ± 6	5.7 ± 0.1
	II)	$(3.6 \pm 0.1) \times 10^6$	-37.5 ± 0.1	70 ± 3	-107 ± 3	8.5 ± 0.2
50 mM	I)	$(2.4 \pm 0.5) \times 10^7$	-42.3 ± 0.5	-231 ± 50	189 ± 50	5.5 ± 0.5
	II)	$(1.2 \pm 0.2) \times 10^6$	-34.7 ± 0.4	69 ± 30	-104 ± 30	7.3 ± 0.2
100 mM	I)	$(8.1 \pm 1.2) \times 10^6$	-39.5 ± 0.4	-216 ± 20	177 ± 20	5.5 ± 0.8
	II)	$(3.1 \pm 0.5) \times 10^5$	-31.4 ± 0.4	65 ± 10	-96 ± 10	6 ± 3

* I) first binding event, II) second binding event

Table S2. Thermodynamic parameters for the binding of Asyn to usGNPs. Given uncertainties are from data fitting.

NaCl	*	K_a (M^{-1})	ΔG ($kJmol^{-1}$)	ΔH ($kJmol^{-1}$)	-TΔS ($kJmol^{-1}$)	n
0 mM	I)	$(1.5 \pm 0.2) \times 10^7$	-41.0 ± 0.3	-195 ± 10	154 ± 10	2.4 ± 0.1
	II)	$(8.3 \pm 0.4) \times 10^5$	-34 ± 2	-48 ± 2	14 ± 4	12.7 ± 0.3
10 mM	I)	$(1.7 \pm 0.4) \times 10^7$	-41 ± 2	-173 ± 9	132 ± 11	2.4 ± 0.1
	II)	$(7.0 \pm 0.5) \times 10^5$	-33 ± 2	-42 ± 2	9 ± 4	12.2 ± 0.4
200 mM	**	$(6.6 \pm 0.8) \times 10^5$	-33.2 ± 0.3	-182 ± 20	149 ± 20	2.2 ± 0.2

* I) first binding event, II) second binding event
** one-site binding model

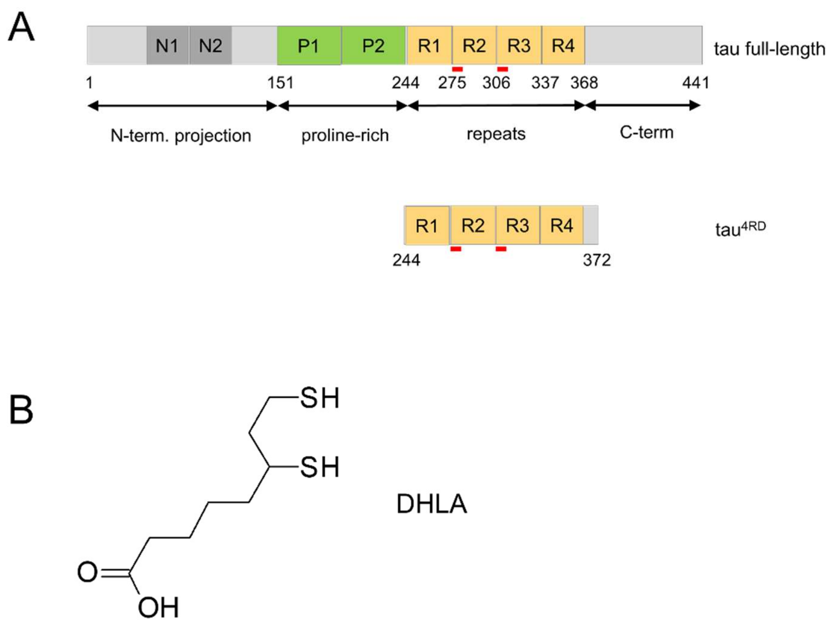

Figure S1. *Molecules used in the study*. A) Domain organization of tau full-length and tau^{4RD}; the position of the hexapeptide motifs is indicated by red bars. B) Structure of dihydrolipoic acid (DHLA).

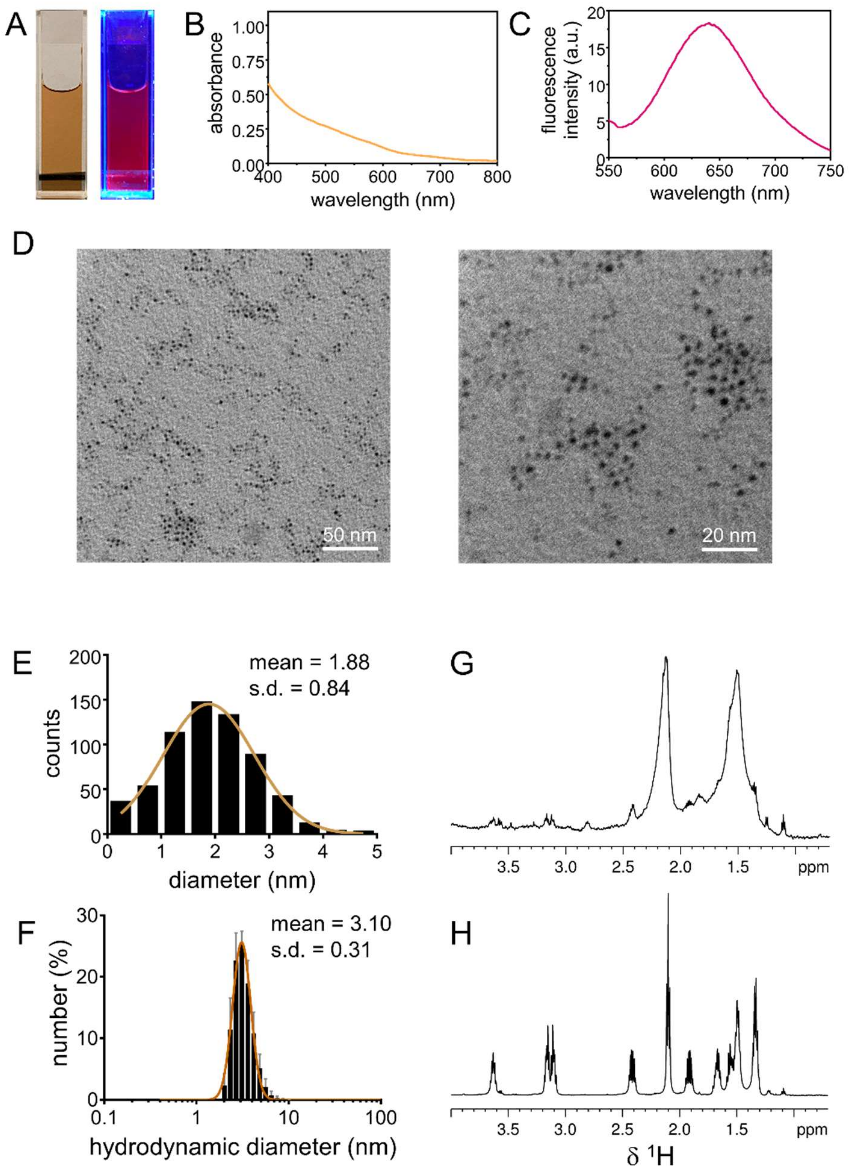

Figure S2. *Characterization of synthesized ultrasmall gold nanoparticles*. A) Colloidal solution of usGNPs visualized under daylight (left) and UV lamp (right). B) The absence of a surface plasmon resonance band in the visible light absorption spectrum is consistent with the ultrasmall size. C) Fluorescence emission spectrum (λ_{ex} = 530 nm, λ_{max} = 640 nm). D) Representative TEM images. E) Diameter distribution histogram derived from TEM micrographs; continuous line is the best-fit Gaussian curve. F) Hydrodynamic diameter distribution as determined from dynamic light scattering; continuous line is the best-fit Log-Gaussian curve. G) The ^{1}H-NMR spectrum of DHLA-capped usGNPs displays broad, poorly resolved signals representing surface-bound DHLA; line broadening results from restricted mobility of the ligand compared to its free state (panel H) and frequency shifts originate from magnetic interactions near the metal surface.[7]. H) ^{1}H-NMR spectrum of the unbound capping ligand.

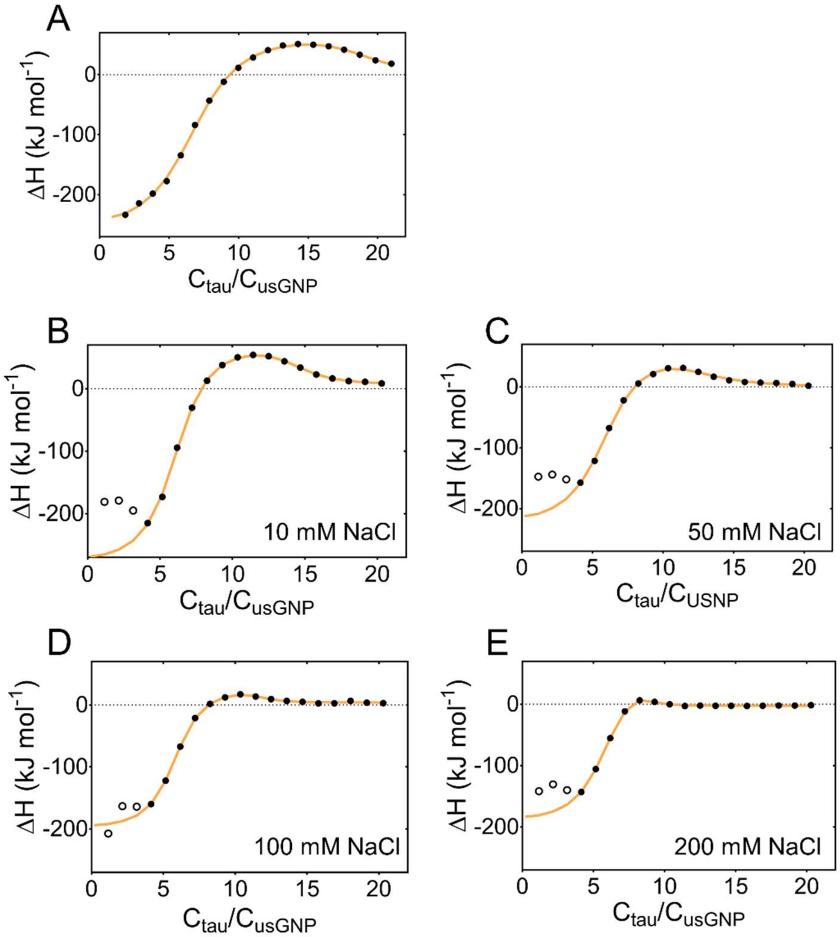

Figure S3. *Ionic strength-dependent thermal response for the interaction between tau^{4RD} and usGNPs*. Isothermal titration calorimetry data obtained on titrating tau^{4RD} into usGNPs, in the presence of A) 0 mM, B) 10 mM, C) 50 mM, D) 100 mM, and E) 200 mM NaCl. All panels display integrated enthalpy plots. Orange lines are best-fit curves based on a two-sets-of-sites binding model; data displayed as empty circles were excluded from fitting. The fitted parameters for A-D are reported in Table S1. For dataset E, no binding parameters are reported as the fitting was unstable.

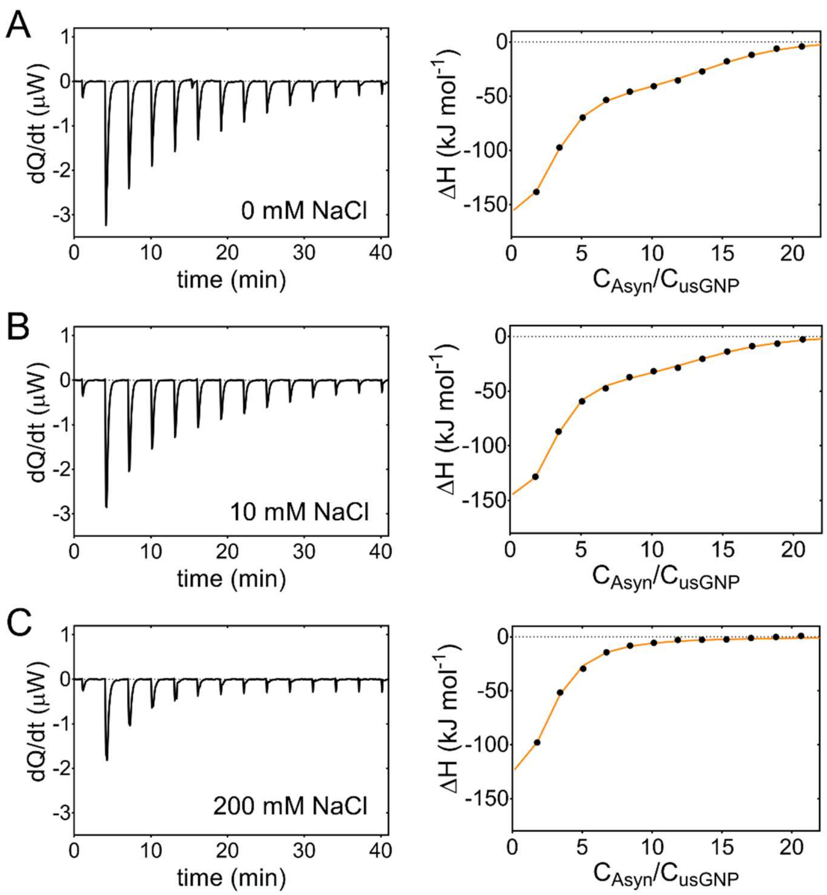

Figure S4. *Isothermal titration calorimetry on α-synuclein/usGNPs*. ITC data obtained on titrating α-synuclein (Asyn) into usGNPs, in the presence of A) 0 mM, B) 10 mM, and C) 200 mM NaCl. All panels display heat flow (left) and integrated enthalpy plots (right). Orange lines are best-fit curves based on (A,B) two-sets-of-sites or (C) one-set-of-sites binding models. The corresponding binding parameters are reported in Table S2.

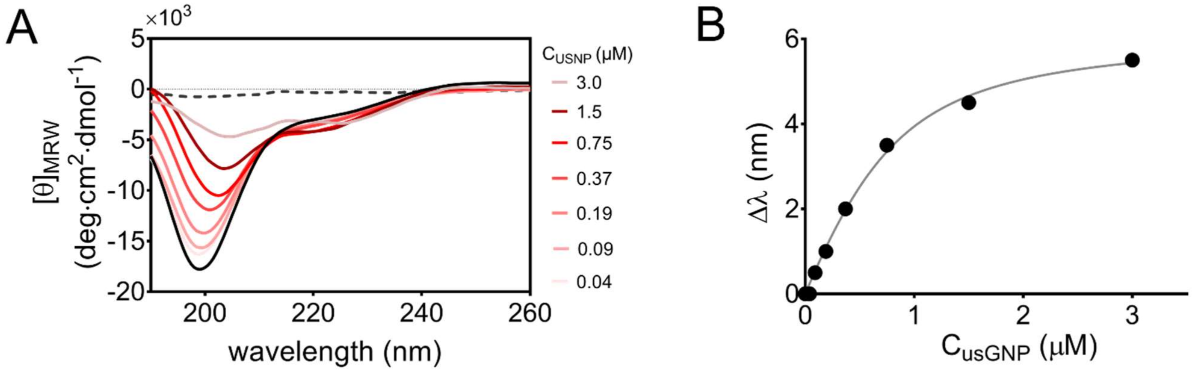

Figure S5. *Secondary structure perturbations.* A) Far-UV CD spectra measured on 6 μM tau^{4RD} in the presence of usGNPs at the indicated concentrations; black continuous line refers to tau^{4RD} alone, dashed line refers to usGNPs alone. B) Concentration dependence of the position of the CD peak with largest ellipticity (ca. 200 nm); solid line corresponds to the best-fit hyperbolic curve.

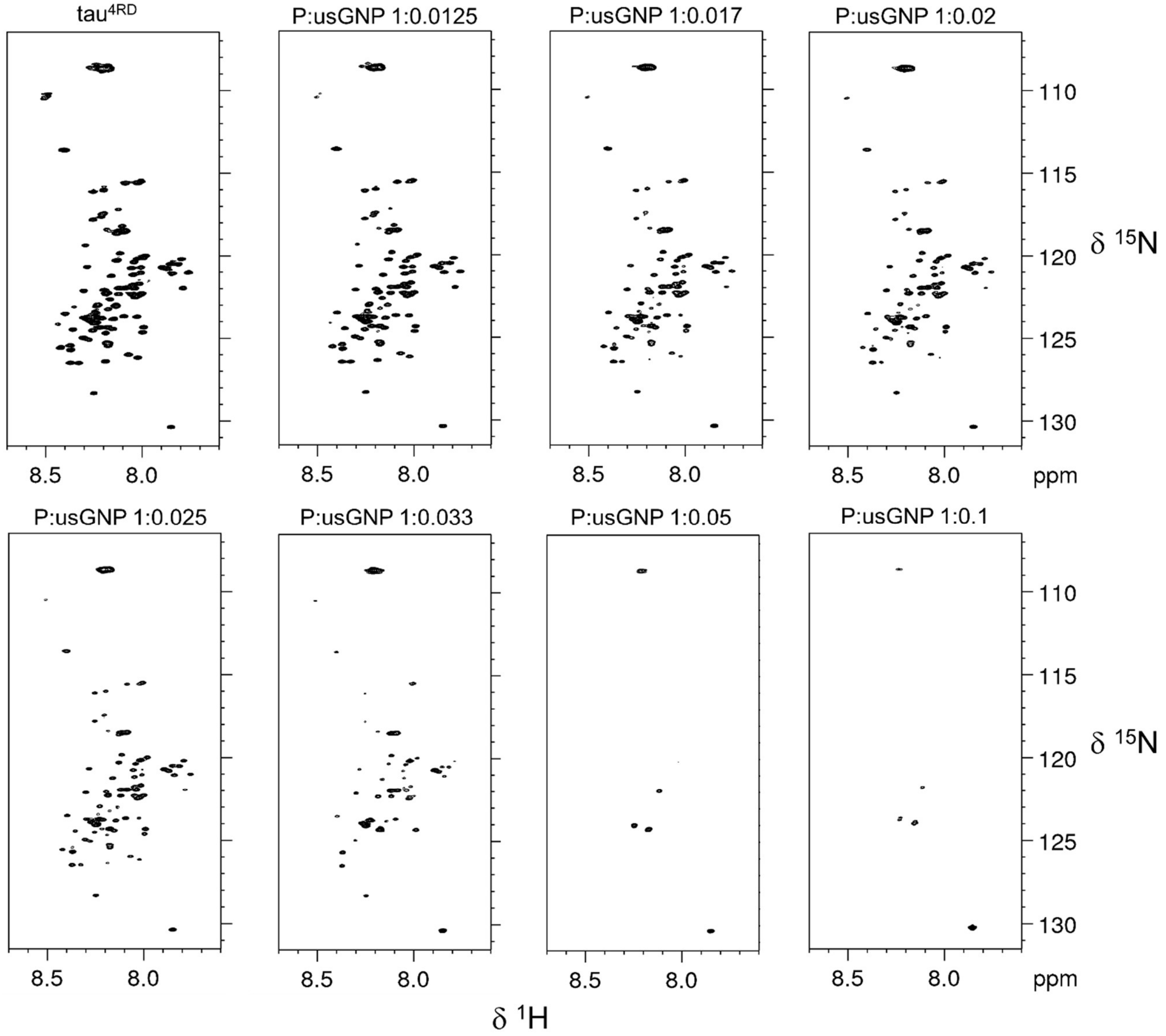

Figure S6. *Protein-observed NMR spectra.* HN-HSQC spectra of tau^{4RD} in the absence (top left) or presence of usGNPs at increasing concentration; the spectral region of side chain amide signals is excluded for better visualization.

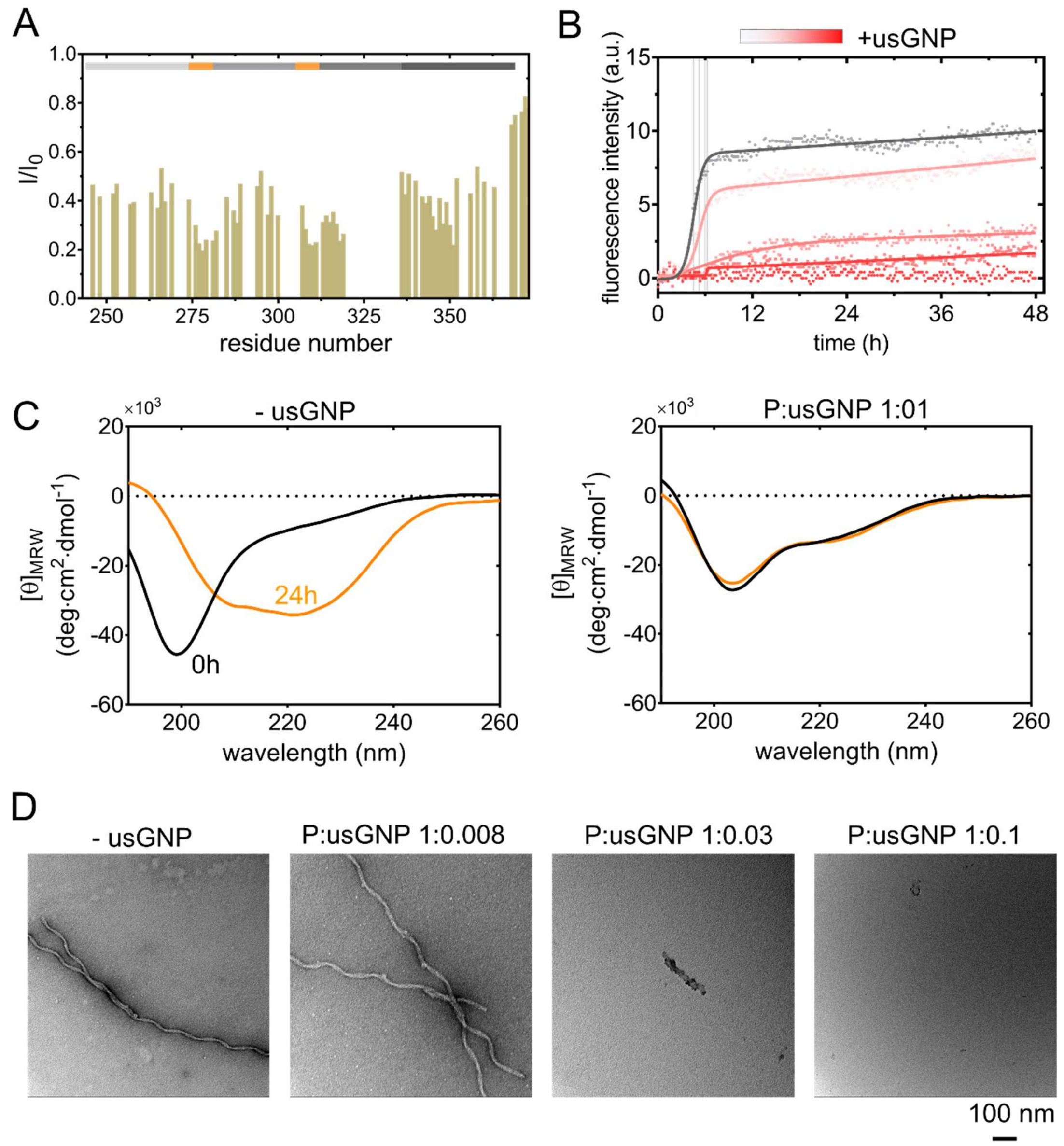

Figure S7. *Binding and aggregation experiments at high ionic strength.* A) Residue-specific HSQC-peak intensity versus residue number; peak intensities were measured on tau^{4RD} in the absence (I_0) or presence (I) of usGNPs at a molar ratio P:usGNPs = 1:0.02; only isolated peaks were included in the analysis; the protein domain organization is shown on top. B) Aggregation kinetics monitored by ThT fluorescence; measurements were performed on tau^{4RD} in the absence (grey dots) or presence (light-to-dark red dots) of usGNPs (P:usGNP molar ratios 1:0, 1:0.002, 1:0.004, 1:0.03, 1:0.1); solid lines correspond to the best-fit curves; vertical grey lines indicate transition midpoints. C) Far-UV CD spectra acquired on tau^{4RD} after 0 h (black) and 24 h (brown) incubation with heparin, in the absence (left) or presence (right) of usGNPs at the indicated molar ratio. Samples were prepared with 100 mM NaCl (NMR, ThT assay) or NaF (CD). D) Representative TEM images of tau^{4RD} samples after 48 h incubation in aggregating conditions and with 100 mM NaCl, in the absence (left panel) or presence (remaining panels) of usGNPs.

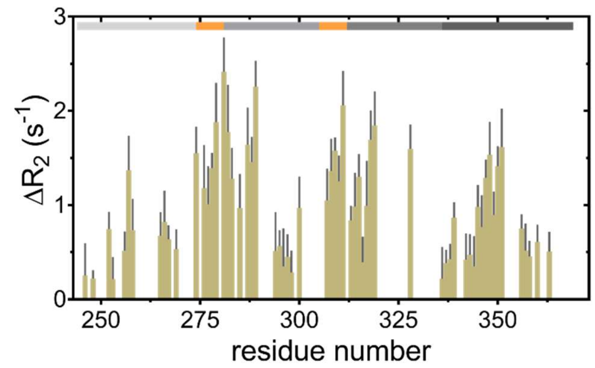

Figure S8. ^{15}N-ΔR_2 (CPMG field = 595 Hz) for 200 μM [^{15}N]tau^{4RD} in the presence of 2 μM usGNPs, 10 mM NaCl, at a spectrometer frequency of 700 MHz. Grey bars are errors derived from exponential fitting of experimental data. Protein domain organization is schematically depicted on the top.

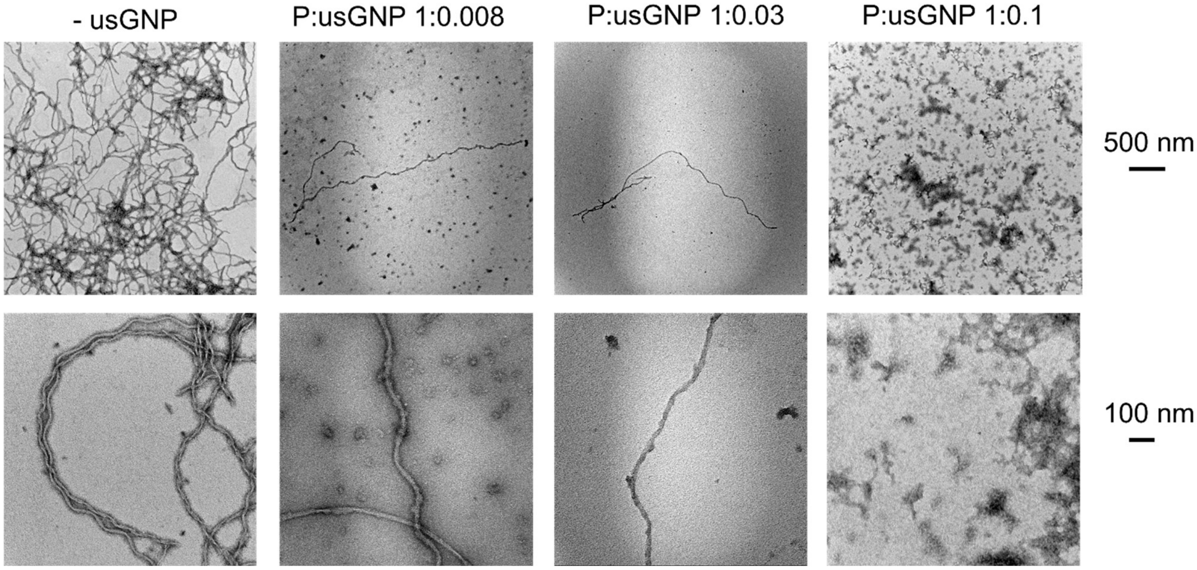

Figure S9. Representative TEM images of tau^{4RD} samples after 48 h incubation in aggregating conditions, in the absence (left panel) or presence (remaining panels) of usGNPs; scale bar for all images in the top (bottom) panels is 500 (100) nm.

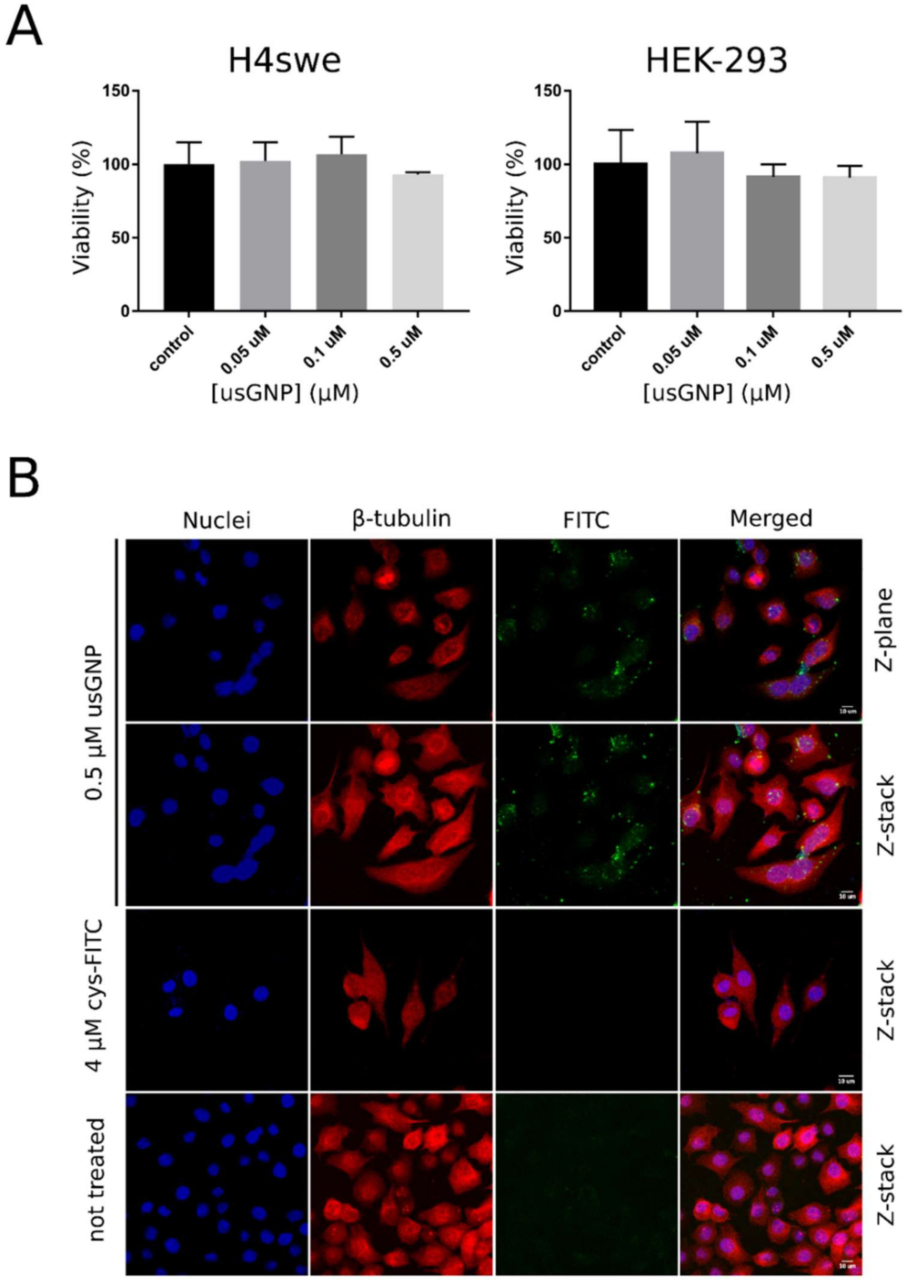

Figure S10. *Biocompatibility and internalization of usGNPs*. A) Cell viability measured by MTT proliferation assay in H4swe (left) and HEK-293 (right) adherent cells treated for 72 h with different concentrations of usGNPs. The results are shown as mean ± S.D. of three independent experiments. One-way ANOVA and Dunnett's multiple comparisons test revealed no statistically relevant differences between the treated samples and the control. B) Representative confocal microscopy images of H4swe cells after 48 h of treatment with 0.5 µM usGNPs (single Z-plane and Z-stack), 4 µM free cysteamine-FITC, and control (not treated). β-tubulin (cytoskeleton) is stained in red; cell nuclei are stained with Hoechst 33342 (blue). Scale bars are 10 µm.

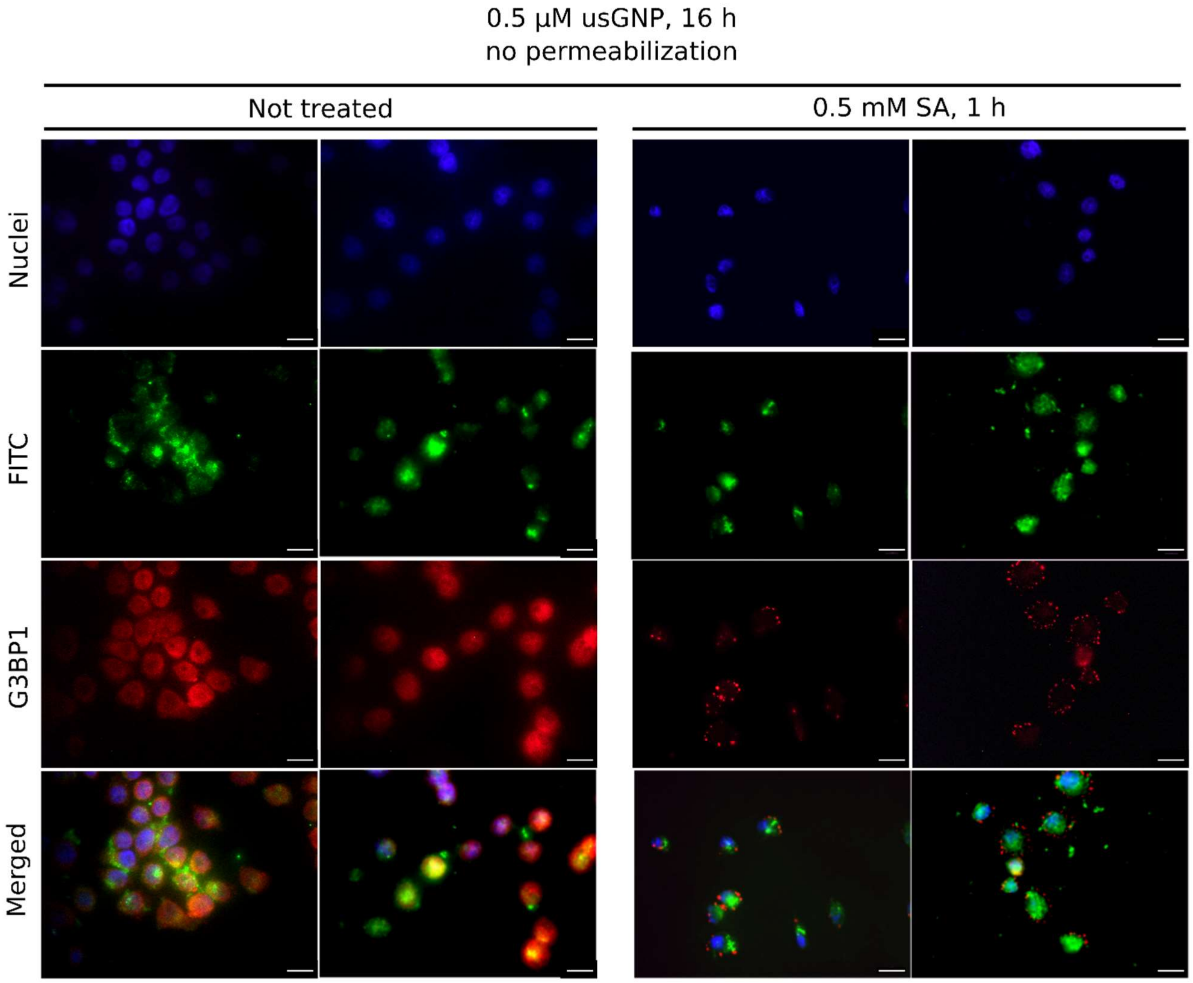

Figure S11. Representative fluorescence microscopy images of unpermeabilized H4-swe cells treated for 16 h with 0.5 µM FITC-usGNPs and subsequently incubated for 1 h in the absence (left) or in the presence (right) of 0.5 mM sodium arsenite (SA). Stress granule marker G3BP1 is stained in red and nuclei in blue (DAPI). Scale bars: 20 µm.

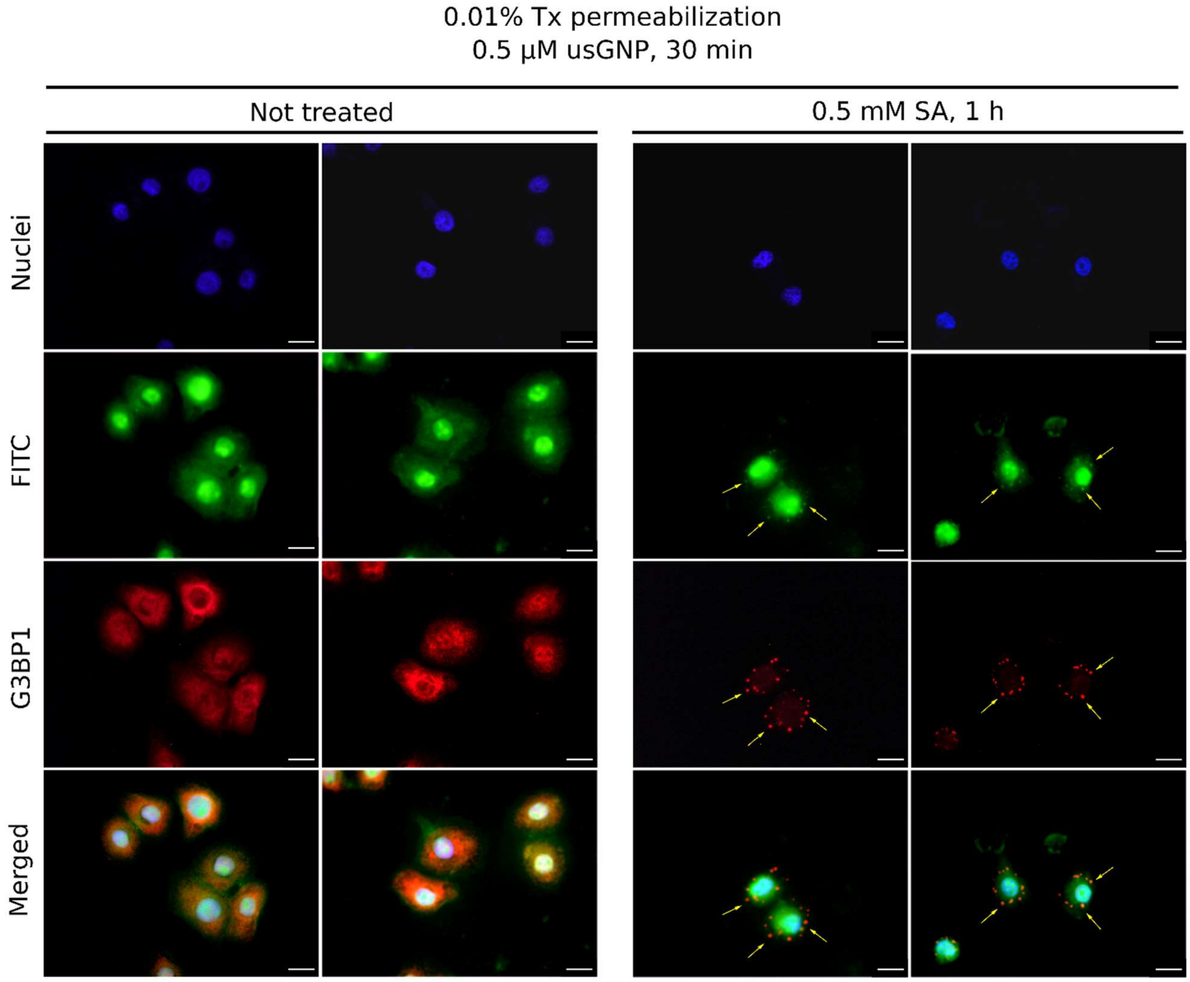

Figure S12. Representative fluorescence microscopy images of H4-swe cells, not treated (left) or treated with 0.5 mM SA (right), incubated for 30 min with 0.5 µM FITC-usGNPs after membrane permeabilization with 0.01% Triton-X100. Stress granule marker G3BP1 is stained in red and nuclei in blue (DAPI). Colocalization of usGNPs with SGs is indicated by the yellow arrows. Scale bars: 20 µm.

REFERENCES

1. Munari, F.; Barracchia, C.G.; Franchin, C.; Parolini, F.; Capaldi, S.; Romeo, A.; Bubacco, L.; Assfalg, M.; Arrigoni, G.; D'Onofrio, M. Semisynthetic and Enzyme-Mediated Conjugate Preparations Illuminate the Ubiquitination-Dependent Aggregation of Tau Protein. *Angew. Chem. Int. Ed Engl.* **2020**, *59*, 6607–6611, doi:10.1002/anie.201916756.
2. Tira, R.; De Cecco, E.; Rigamonti, V.; Santambrogio, C.; Barracchia, C.G.; Munari, F.; Romeo, A.; Legname, G.; Prosperi, D.; Grandori, R.; et al. Dynamic Molecular Exchange and Conformational Transitions of Alpha-Synuclein at the Nano-Bio Interface. *Int. J. Biol. Macromol.* **2020**, *154*, 206–216, doi:10.1016/j.ijbiomac.2020.03.118.
3. Shang, L.; Brandholt, S.; Stockmar, F.; Trouillet, V.; Bruns, M.; Nienhaus, G.U. Effect of Protein Adsorption on the Fluorescence of Ultrasmall Gold Nanoclusters. *Small* **2012**, *8*, 661–665, doi:https://doi.org/10.1002/smll.201101353.
4. Shang, L.; Azadfar, N.; Stockmar, F.; Send, W.; Trouillet, V.; Bruns, M.; Gerthsen, D.; Nienhaus, G.U. One-Pot Synthesis of Near-Infrared Fluorescent Gold Clusters for Cellular Fluorescence Lifetime Imaging. *Small* **2011**, *7*, 2614–2620, doi:https://doi.org/10.1002/smll.201100746.
5. Haiss, W.; Thanh, N.T.K.; Aveyard, J.; Fernig, D.G. Determination of Size and Concentration of Gold Nanoparticles from UV−Vis Spectra. *Anal. Chem.* **2007**, *79*, 4215–4221, doi:10.1021/ac0702084.
6. Beckwith, M.A.; Erazo-Colon, T.; Johnson, B.A. RING NMR Dynamics: Software for Analysis of Multiple NMR Relaxation Experiments. *J. Biomol. NMR* **2021**, *75*, 9–23, doi:10.1007/s10858-020-00350-w.
7. Ruks, T.; Beuck, C.; Schaller, T.; Niemeyer, F.; Zähres, M.; Loza, K.; Heggen, M.; Hagemann, U.; Mayer, C.; Bayer, P.; et al. Solution NMR Spectroscopy with Isotope-Labeled Cysteine (^{13}C and ^{15}N) Reveals the Surface Structure of L -Cysteine-Coated Ultrasmall Gold Nanoparticles (1.8 Nm). *Langmuir* **2019**, *35*, 767–778, doi:10.1021/acs.langmuir.8b03840.
